# The Tishchenko reaction mediated by organo-f-complexes: the myths and obstacles

**DOI:** 10.1039/d4ra01824a

**Published:** 2024-06-05

**Authors:** Aditya L. Shinde, Moris S. Eisen, Tapas Ghatak

**Affiliations:** a Advanced Catalysis Facility, Department of Chemistry, School of Advanced Sciences, Vellore Institute of Technology Vellore-632014 Tamil Nadu India tapaschem@gmail.com tapas.ghatak@vit.ac.in; b Schulich Faculty of Chemistry, Technion – Israel Institute of Technology Technion Israel

## Abstract

For over a century, the Tishchenko reaction has been a valuable technique for synthesizing esters from aldehydes, serving a variety of applications in different domains. Beyond the remarkable advances in organoactinide and organolanthanide chemistry over the past two decades, there has been a significant increase in the research of the electrophilic d0/fn chemistry of organoactinide and organolanthanide compounds due to the captivating interplay between their structure and reactivity, and their exceptional performance in various homogeneous catalytic processes. The remarkable influence of ligand design, both in terms of steric hindrance and electronic properties, on the catalytic activity of organo-f-element complexes in organic transformations is well-established. However, the traditional view was that the significant oxophilicity of actinide and lanthanide complexes makes them unfavorable for reactions involving oxygen because of catalytic poisoning and their applications have been relatively limited, primarily focused on hydroalkoxylation, small-molecule activation, and cyclic ester polymerization. This review dissects the intricate interplay between ligand design and catalytic activity in actinide and lanthanide complexes, specifically in the context of the Tishchenko esterification.

## Abstract 2

For over a century, the Tishchenko reaction has been a valuable technique for synthesizing esters from aldehydes, serving a variety of applications in different domains.

## Graphical abstract

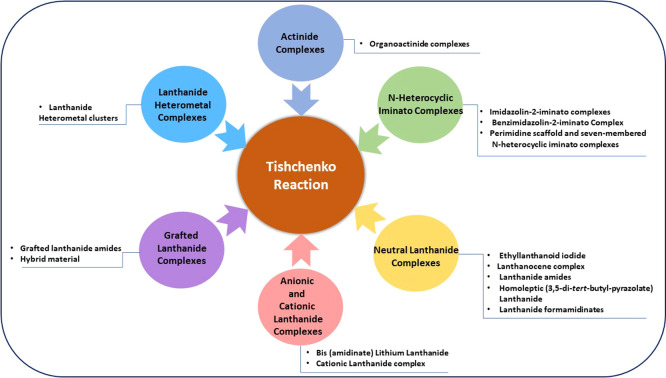

## 1. Introduction

**Scheme 1:**
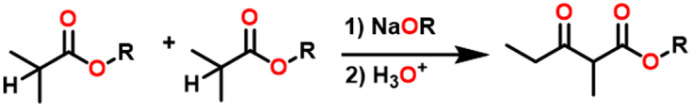
Claisen condensation reaction.

Esters are versatile chemical compounds with applications in a variety of industries like plastics, solvents, fragrances, flavors, and even pharmaceuticals and agriculture. Traditionally, esters were synthesized by reacting carboxylic acids and alcohols with catalysts like acids^1^ or metals,^2^ generating the desired ester and water. However, this process often necessitates specific techniques or excess starting materials to overcome the low product yield owing to the equilibrium favoring both the ester and byproducts.^3,4^ Unfortunately, using these traditional methods results in significant waste and underutilizes the starting materials, raising concerns about both impacts on the environment and efficiency. Back in 1887, a pioneering chemist named Rainer Ludwig Claisen discovered a remarkable reaction: the Claisen condensation.^5,6^ This ingenious process forges carbon–carbon bonds, bridging esters or other carbonyl compounds together. Ever since its discovery, the Claisen condensation has captivated chemists worldwide, inspiring countless investigations and innovations (Scheme 1).

**Scheme 2:**
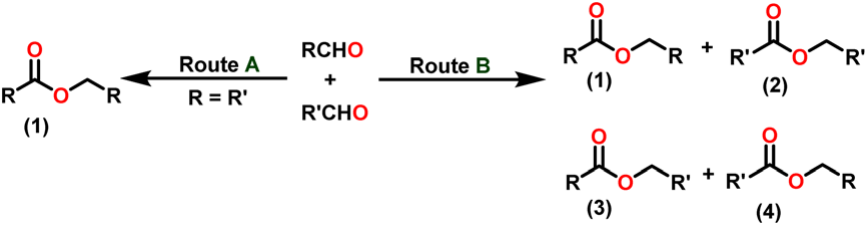
General Tishchenko reaction.

Claisen pioneered the pathway for aldehyde esterification by revealing their dimerization potential in the presence of sodium alkoxides. Building on this basis, Tishchenko established the process as an overall strategy, using various metal alkoxides (Al or Mg) to catalyze the reaction across an additional range of aldehydes.^3^ The Tishchenko reaction, as demonstrated by routes A and B in Scheme 2, is versatile in terms of starting materials. Route A uses two similar aldehydes to form a symmetrical ester (1), while route B allows the combination of different aldehydes to yield a mixture of products (1–4).^3,4^

**Scheme 3:**
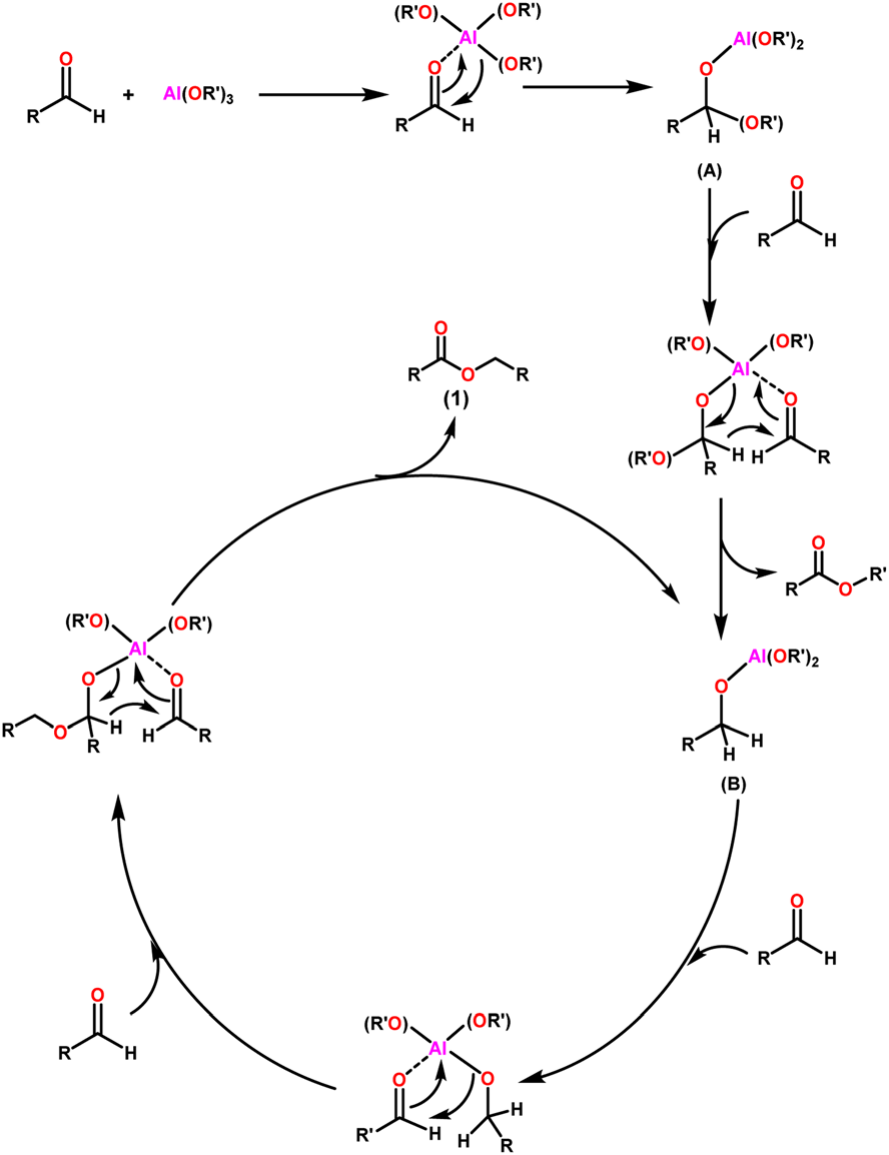
General mechanism of Claisen–Tishchenko reaction.

The widely accepted mechanism of the Claisen–Tishchenko reaction begins with the aldehyde coordination to the Lewis acidic aluminium alkoxide catalyst (as shown in Scheme 3). The coordinated hemiacetal (A) is formed by shifting an alkoxide group to the aldehyde. Another aldehyde molecule then bonds to the central aluminium atom. The hydride transfer occurs from the hemiacetal to the new aldehyde, releasing a mixed ester, and the reduced aldehyde now acts as a new alkoxide ligand for the aluminium. This process is repeated as a third aldehyde molecule interacts with the new aluminium complex (B), transferring the previously created alkoxide group to the aldehyde. After the cycle is completed, symmetrical ester (1) is cleaved, allowing the catalyst to start another cycle (Scheme 3).

Mechanistically, the Tishchenko reaction is part of a family of reactions involving alkoxide-driven transformations of aldehydes, including Cannizzaro,^7,8^ Meerwein–Ponndorf–Verley,^9^ and Oppenauer oxidation.^10^ A diverse range of catalysts, both homogeneous and heterogeneous can be utilized to promote the Tishchenko reaction. The Tishchenko reaction has been catalyzed by many metal complexes^3,4,6,11^ and solid-phase catalysts.^12^ The bidentate aluminum complexes like sodium organoaluminate complex,^13^ aluminum alkoxide complexes,^14^ and *N*,*O*-bidentate aluminum complexes^15^ a very potent catalyst considerably aided the Tishchenko reaction. Other catalysts employed include alkali and alkaline earth metal oxides (including Grignard-type compounds),^16,17^ lanthanoids,^6,18^ and transition metals.^19,20^ The development of heterogeneous catalysts for the Tishchenko reaction has mostly focused on alkaline earth metal oxides and different types of alumina. However, these catalysts tend to be very slow or only show reactivity in extremely stringent reaction conditions.

**Scheme 4:**
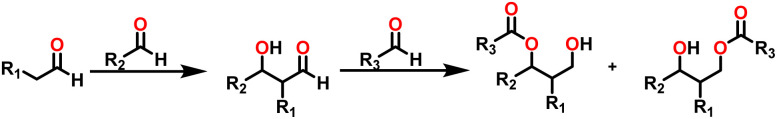
General aldol Tishchenko reaction.

**Scheme 5:**
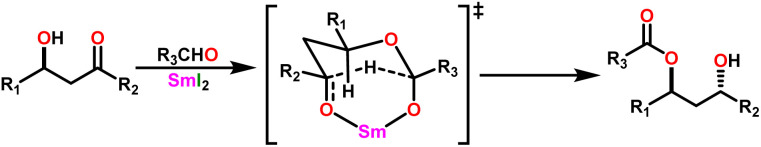
General Evans–Tishchenko reaction.

By the mid-twentieth century, interest in the Tishchenko reaction had grown because of the emergence of different variations of the Tishchenko reaction, and substantial growth in this topic in recent years. A well-known process called the aldol reaction bridges two molecules that contain carbonyl to form a new C–C bond.^21,22^ A β-hydroxy ketone was formed, regardless of whether the two are aldehydes or a combination of aldehydes and ketones in the aldol reaction. The Tishchenko reaction has led to two famous modifications, each adding a distinctive twist to its core principle: the aldol Tishchenko reaction,^23,24^ which incorporates it with the classical C–C bond formation of the aldol reaction, and the Evans–Tishchenko reaction^25,26^ which was known for its diastereoselective reduction of β-hydroxy ketones. The aldol Tishchenko reaction was a two-step reaction where the first step comprised an aldol reaction employing two different aldehydes, and the second step comprised the subsequent coordination of another aldehyde, which was then followed by a Tishchenko-type hydride transfer (Scheme 4). In Evans–Tishchenko reaction catalytic samarium iodide was employed to yield 1,3-antidiol monoesters^3,27–29^ (Scheme 5). This reaction can take place with enolizable aldehydes (aldehyde trimerization), or it can take place with ketones (formation of 1,3-diol monoesters). The Evans–Tishchenko reaction, a mild and selective approach for the synthesis of 1,3-*anti* diol structures, was employed for the total synthesis of natural products.^30–42^ Other options for ester synthesis such as Favorskii,^43^ Fischer,^44,45^ Steglich,^46^ and Yamaguchi^47^ reactions were available, but their limitations made them unsuitable for an industrial-applications. From an atom economy perspective, Tishchenko reaction-based ester synthesis outperforms typical methods, which often yield undesirable leaving groups and side products that do not contribute to a final product. The Tishchenko reaction has the enticing technical advantage of being able to be carried out by aldehyde as a reactant and the solvent and pushed almost to completion, without the need for any additional purification processes. Modifying the parameters that govern the reaction conditions (temperature, pressure, solvent, and catalyst) can exert a significant influence on the product distribution of the Tishchenko reaction. The emphasis on the coordinating metal and its surrounding ligands led to the development of diverse catalysts for the Tishchenko reaction. While transition metal catalysts such as [Cp_2_ZrH_2_] (5),^48^ [H_2_Ru(PPh_3_)_2_] (6),^49^ and K_2_[Fe(CO)_4_] (7)^50^ exhibited efficacy in Tishchenko reactions, they were not without their limitations.

Inner transition organometallic compounds have emerged as promising alternatives to traditional transition metal complexes, owing to a need for novel catalysts and the unique properties of actinides and lanthanides. Recent research has focused significant emphasis on the development of organoactinide and organolanthanide complexes as catalysts for Tishchenko reactions. This increased interest stems from their advantageous properties, including high Lewis acidity, high coordination numbers, relatively polar metal–ligand bonding, a tunable coordination sphere around the metal center, a plethora of coordination geometries, and environmental friendliness. The steric and electronic properties of auxiliary ligands employed in organo-f-element complex-mediated organic transformations have a significant influence on the catalytic activity exhibited by the organo-f-element complex systems.^51–53^ The distinctive reactivities of organoactinide and organolanthanide compounds often complement those observed in the main group and, transition metal. An example of this is the fact that, unlike the *anti*-Markovnikov addition products generated by transition metal precatalysts, actinide complexes showed Markovnikov selectivity when hydrothiolating terminal alkynes.^54,55^ The prevalence of unique oxidation states (+3 in lanthanides and +4 in early actinide (Th)) precludes the conventional catalytic routes of oxidative addition and reductive elimination commonly observed in transition-metal complexes. Consequently, organoactinide and organolanthanide-induced transformations are primarily characterized by olefin insertion and metathesis, respectively.^56–60^ The strong affinity of actinide and lanthanide centers for oxygen-containing substrates posed a significant challenge in organo-f-chemistry. The formation of stable and unreactive oxygen–metal bonds hinders the transformation of these substrates, contributing to the historical lag observed in this field compared to d-block metal complexes. While actinide could be useful for a variety of applications, its relationship with nuclear weapons is likely to affect public opinion and research priorities.^61–66^ Furthermore, the challenges of obtaining sufficient actinide starting materials and addressing safety concerns have also contributed to the historical lag in progress.^66–68^ This delay can also be attributed to the limited research efforts dedicated to this field.

Despite their enormous capabilities, organo-f-element complexes had limited applications in oxygen-containing substrate reactions. To date, only a handful of transformations, including hydroalkoxylation,^69–72^ hydrosilation,^73–75^ hydrophosphination,^69,76^ hydroamination,^69,77–80^ alcohol insertion into carbodiimides,^72,81,82^ small-molecule activation,^83–86^ and cyclic ester polymerization^51,87–89^ catalyzed by organo-f-element complexes have been documented. Historically, the application of organo-f-element complexes in aldehyde-based reactions was limited due to concerns about catalyst poisoning arising from strong oxygen-f-block metal interactions. However, in recent years, there has been a significant increase in the development of organo-f-element catalysts capable of effectively catalyzing the Tishchenko reaction.^51,82,90–97^

In this review, various organoactinides and organolanthanide catalysts such as cyclopentadienyl-actinide complexes,^94,98^ imidazolin-2-iminato (Im^R^N) actinide complexes,^53,99–101^ N-heterocyclic iminato actinide complexes,^102^ ethyllanthanoid iodide,^103^ lanthanocene complexes,^104^ lanthanide amide complexes,^6,105^ homoleptic rare-earth pyrazolate complexes,^18^ lanthanide formamidinates,^106,107^ bis(amidinate)lithium lanthanide complexes,^108^ cationic lathanide complex,^157^ grafted lanthanide amide complexes,^109^ hybrid material [SBA-15]Sm[N(SiMe_3_)_2_]_*x*_ (ref. 110) and heterometal clusters^111^ employed in the Tishchenko reaction had been explained.

In earlier works, the stoichiometric and catalytic chemistry of transition metals in the Tischenko reaction and similar reactions have been discussed. However, at this time, there is no review paper accessible that includes the only organo-f-elements catalyzed Tischenko reaction. Following an introductory section, this review is structured as follows: catalytic Tischenko reaction mediated by (i) organoactinides and (ii) organolanthanides. Lastly, we share our Quo Vadis viewpoint, ask some thought-provoking questions, and provide our viewpoint on the future of this area.

## 2. Actinide complexes catalysed Tishchenko reactions

Over the last two decades, both anionic and neutral organoactinide complexes have been the subject of intense research for their potential to function as catalysts in a variety of organic reactions. Some of these reactions were polymerization of alkenes,^93,112^ oligomerization,^113,114^ intermolecular hydroamination,^69,77,78,115^ hydrosilylation of terminal alkynes,^73,116^ and hydrophosphination,^69^*etc.* However, because of the strong oxophilicity of the actinide complexes (208.0 kcal mol^−1^ for ThO and 181.0 kcal mol^−1^ for UO), all substrates containing oxygen atoms, especially aldehydes, were eliminated and the predicted poor activity of these complexes was attributed to the foreseeable oxygen–actinide interaction.

**Scheme 6:**
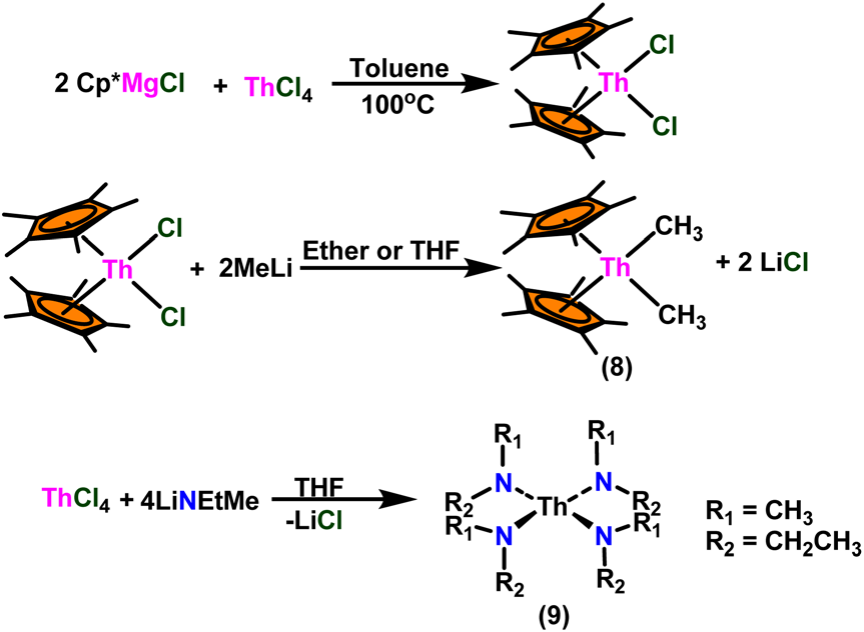
Synthesis of Cp*ThMe_2_(8) and Th(NEtMe)_4_(9).

Andrea *et al.* described that organoactinide-mediated catalytic Tishchenko reactions involving two identical or different aldehydes resulted in the formation of symmetric (1 and 2) or asymmetric (3 and 4) esters, respectively (Scheme 6). In order to provide a suitable mechanism for the Tischenko reaction and to imply the process's application, two organoactinide complexes, Cp*_2_ThMe_2_(8) and Th(NEtMe)_4_(9), were investigated. By substituting an alkoxo ligand (OR) for one of the methyl groups in Cp*_2_ThMe_2_, the hydrogenolysis efficiency was reduced by a factor of 4000. Intriguingly, substrate activity was determined by the closeness of phenyl group substituents to a metal center. The reduced activity of the substrate with *ortho* substituent relative to *para* isomers may be ascribed to steric hindrance. The two organoactinide complexes (8 and 9) were discovered as highly to moderately active (yields 85–65%) in the catalytic dimerization of benzaldehyde and other substituted benzaldehyde to give the corresponding ester with no by-products. In the Tishchenko reaction, tweaking the ratio of reactants allowed to fine-tune the proportions of the four possible products. An intriguing result of the reaction between two different aldehydes was the appearance of four possible isomers. As an illustration, when *p*-tolualdehyde and benzaldehyde reacted, as expected, the less reactive *p*-tolualdehyde participated in the catalytic process to a lesser extent catalytic process, resulting in benzoyl benzoate consistently being the main product. A kinetic and thermodynamic investigation of complex 8 was carried out to propose a plausible mechanism for the reaction and learn about the effects of aldehyde, catalyst, and temperature on the reaction rate.

1 *V* = *k*[catalyst]^1^[aldehyde]^1^

As seen in eqn (1), the reaction had a first-order dependence on both catalyst and aldehyde. According to thermodynamic studies, the energy of activation (*E*_a_), enthalpy of activation (Δ*H*^‡^), and entropy of activation (Δ*S*^‡^) for the rate-determining step revealed 7.16 ± 0.40 kcal mol^−1^, 6.5 ± 0.4 kcal mol^−1^, and −48.8 ± 0.4 eu, respectively. At the rate-determining step, the high negative entropy value indicated a highly ordered transition state. A primary isotopic effect was observed with *k*_H_/*k*_D_ = 2.7, indicating that hydride transfer was involved in the rate-determining step.

**Scheme 7:**
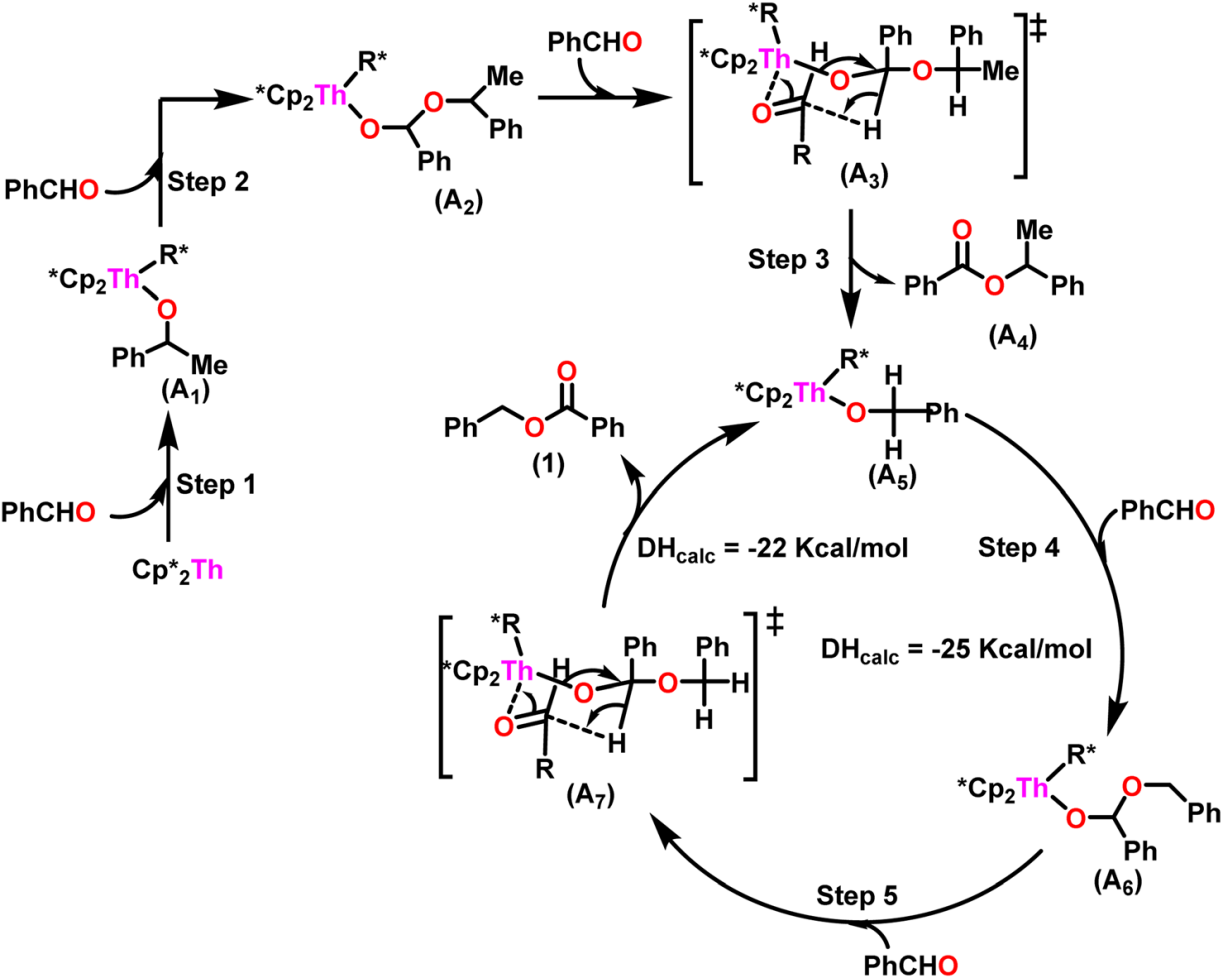
A plausible mechanism for catalytic Tishchenko reaction mediated by organoactinide complex 8.

**Scheme 8:**
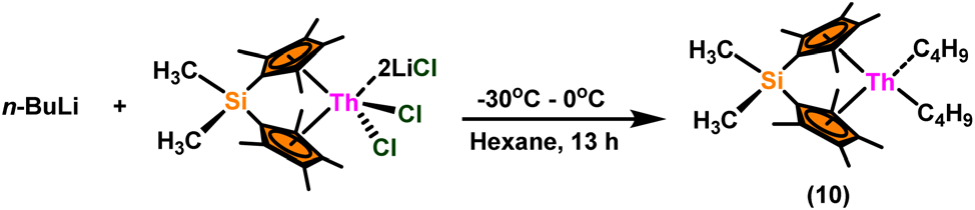
Synthesis of catalyst Me_2_SiCp*_2_ThBu_2_(10).

A plausible mechanism for the Tishchenko reaction was presented in Scheme 7 based on the kinetic and thermodynamic data.^94^ The catalytic cycle commenced with the formation of the metal alkoxy intermediate (A_1_)*via* a four-center transition state initiated by a thermodynamically favorable interaction between two aldehyde molecules and precatalyst 8.^94,117,118^ The complex (A_2_) was formed by the second insertion of an aldehyde into the thorium–alkoxide bond. In a subsequent step, the ester (A_4_) was produced by the metathesis of complex (A_2_) with an additional aldehyde through a six-membered transition state (A_3_). The catalytic insertion of an aldehyde into a thorium alkoxo bond was followed by a hydride transfer reaction with another aldehyde *via* a plausible six-centered transition state, which produced ester (1) and regenerated the active complex (A_5_) (Scheme 7). An actinide-alkoxy bond, long considered to constitute an impasse in the use of actinide complexes as catalysts, was activated during the process. Hence this was the first example of actinide complex-mediated catalytic coupling of aldehyde. The organoactinide complexes 8, 9, and 10 were efficient precatalysts for the chemoselective dimerization of aldehydes, leading to the formation of the esters in moderate to high yields. The corresponding esters formed *via* the reaction of these complexes with an excess of aldehyde in toluene or benzene (aldehyde : catalyst = 100 : 1) at room temperature. Sharma *et al.* proposed a strategy in which a metal–oxygen bond was thermodynamically inserted with a substrate to generate a second metal–oxygen bond with equal bond energies, while entropy being the major parameter controlling the reaction.^98^ In the presence of Argon, Me_2_SiCp′′_2_ThCl_2_–2LiCl–2DME was slowly added to *n*-butyl lithium at −30 °C. The reaction was warmed to 0 °C and stirred for an additional 30 minutes. The condensed pentane was removed with a vacuum, and the orange-brown solids were dried in a vacuum for 13 hours. The solid was collected by cold filtration, and re-crystallization from DME formed a pale yellow solid. *i.e.* (Me_2_SiCp*_2_ThBu_2_) (Scheme 8).^98,119–121^

Enhanced reaction rates were reported when the same dimerization process was conducted at a marginally elevated temperature of 35 °C. According to the results, the rate of dimerization rises as the coordinative unsaturation of the organoactinide complexes utilized in this research increases, with 10 being the highest, followed by 9 and 8, respectively. Kinetic measurements were conducted to enhance comprehension of the impact of auxiliary ligands, as well as the influence of the aldehyde and catalyst on the rate of the reaction. The dimerization of aldehydes promoted by complexes 8 and 10 can be expressed as eqn (2).

2 *ν* = *k*[complex]^1^[aldehyde]^1^

Since complex 10 readily binds to other molecules (highly coordinatively unsaturated), a limiting substrate : catalyst ratio was present to achieve the fastest reaction for which the optimal substrate : catalyst ratio was around 300 to 1. For complexes 8 and 10, at constant concentrations of benzaldehyde and catalyst, similar kinetic dependence was observed on aldehyde and the catalyst over the range of temperature studied (35–95 °C). The activation parameters determined by Arrhenius and Eyring analysis were *E*_a_ = 7.16 kcal mol^−1^, Δ*H*^‡^ = 6.58, kcal mol^−1^, Δ*S*^‡^ = −48.8 eu and *E*_a_ = 3.47 kcal mol^−1^, Δ*H*^‡^ = 2.80 kcal mol^−1^, Δ*S*^‡^ = −65.2 eu for complexes 8 and 10 respectively.^98^ Complex 10 had a higher negative Δ*S*^‡^, indicating a more structured transition state, which facilitated a higher degree of coordinative unsaturation. To evaluate the effect of substituents on the benzene ring while the dimerization reaction was taking place, the thermodynamic parameters (*E*_a_, Δ*H*^‡^, and Δ*S*^‡^) of the rate-determining step (RDS) were measured and calculated for the *meta*- and *para*-substituted benzaldehyde with the electron-withdrawing chlorine and the electron-donating methyl group using complex 10 (Table 1). From Table 1, it can be concluded that the presence of the electron-withdrawing group on the aromatic ring resulted in a slightly lowered *E*_a_ compared to the presence of the electron-donating group on the aromatic ring. For complex 10, a primary isotopic effect was observed using deuterated benzaldehyde with *K*_H_/*K*_D_ = 2.7, revealing that hydride transfer was involved in the RDS.^122^ When complexes 8, 9, and 10 were used, stoichiometric reactions between actinide complexes and benzaldehyde yielded the stoichiometric amounts of esters. Complexes 8, 9, and 10 displayed remarkable catalytic activity in the selective dimerization of aldehydes, resulting in desired esters with excellent chemoselectivity and no by-products. The observed catalytic activity trend was 10 > 9 > 8, maybe due to the differences in coordinative unsaturation around the metal center (Table 2).

**Table 1:** Thermodynamic data of substituted and unsubstituted benzaldehyde during the Tishchenko reaction with precatalyst 10

Substrate	*E*_a_ (kcal mol^−1^)	Δ*H*^‡^ (kcal mol^−1^)	Δ*S*^‡^ (eu)
Benzaldehyde	3.47	2.80	−65.2
*m*-Chlorobenzaldehyde	3.45	2.79	−65.3
*p*-Chlorobenzaldehyde	3.41	2.75	−65.0
*m*-Methylbenzaldehyde	3.90	3.24	−64.4
*p*-Methylbenzaldehyde	4.36	3.70	−64.6

**Table 2:** Dimerization of aldehydes by thorium complexes

S. no.	Catalyst	RCHO [% yield]
1	Cp*_2_ThMe_2_	Ph-[65], *p*-CH_3_-Ph-[25], *m*-CH_3_-Ph-[20], *o*-CH_3_-Ph-[10], *p*-Cl-Ph-[84], *m*-Cl-Ph-[81], *o*-Cl-Ph-[75]
2	Th[NEtMe]_4_	Ph-[85], *p*-CH_3_-Ph-[82], *m*-CH_3_-Ph-[75], *o*-CH_3_-Ph-[55], *p*-Cl-Ph-[97], *m*-Cl-Ph-[96], *o*-Cl-Ph-[95]
3	Me_2_SiCp′′_2_Th(*n*-C_4_H_9_)	Ph-[96], *p*-CH_3_-Ph-[74], *m*-CH_3_-Ph-[94], *o*-CH_3_-Ph-[70], *p*-Cl-Ph-[98], *m*-Cl-Ph-[95], *o*-Cl-Ph-[89], *p*-CN-Ph-[98], *p*-MeO-Ph-[39], *m*-NO_2_-Ph-[98]

**Scheme 9:**
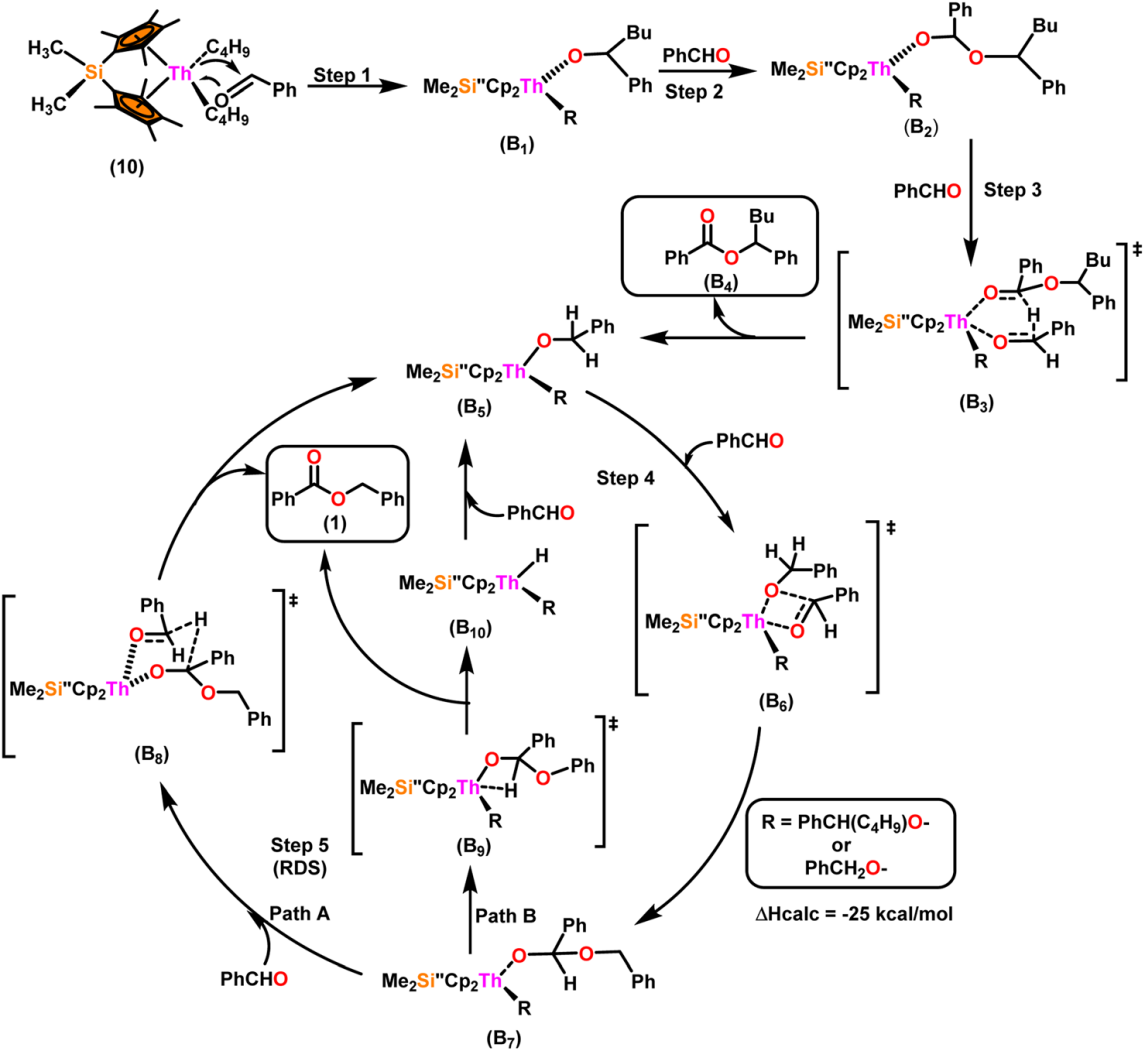
A plausible mechanism for the catalytic dimerization of aldehyde by organoactinide complex 10.

**Scheme 10:**
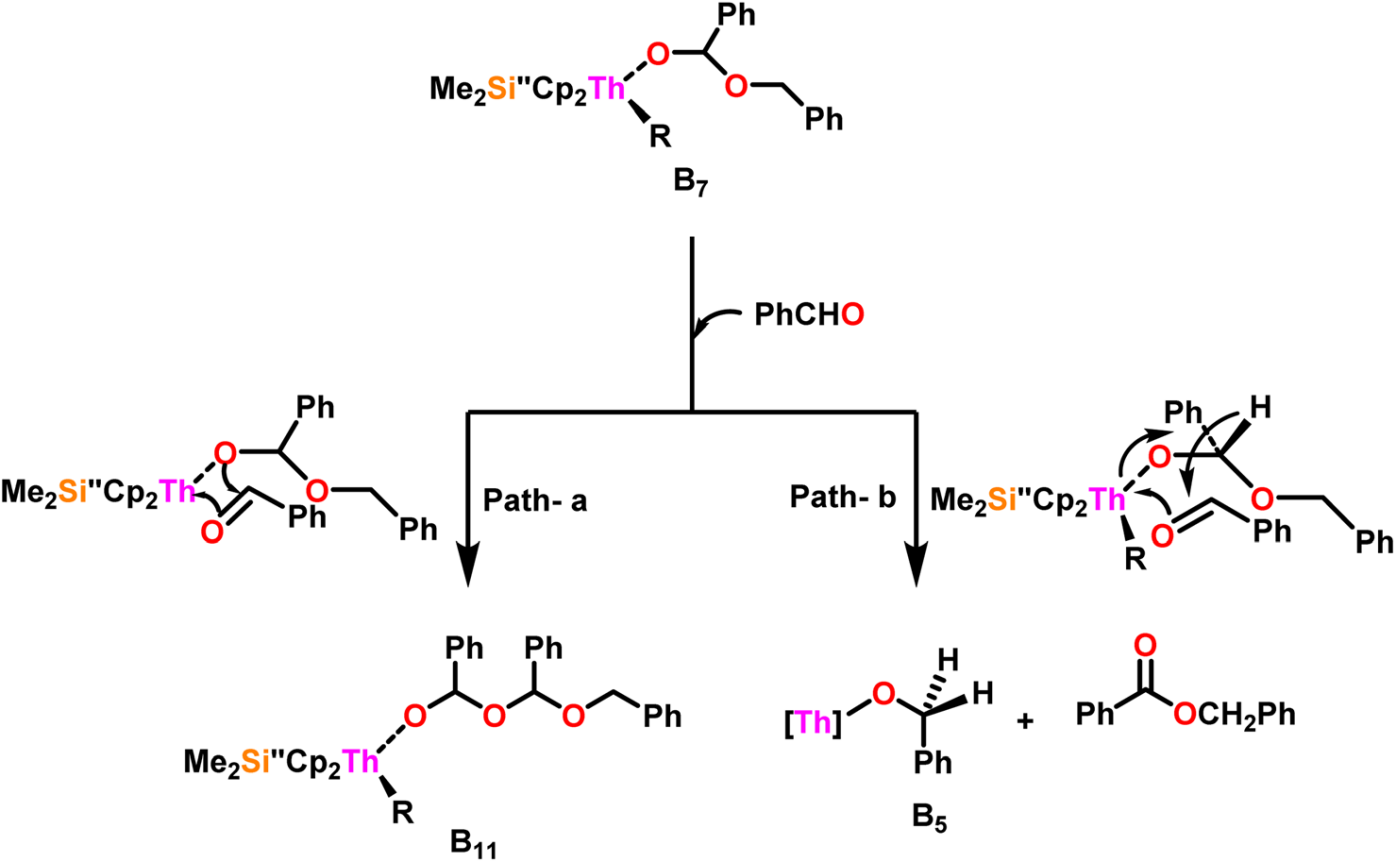
Two possible pathways for the reaction of benzaldehyde with complex B_7_.

Scheme 9 depicts a plausible mechanism for the selective dimerization of aldehydes facilitated by the organothorium complex 10.^98^ The catalytic process was initiated by adding two equivalents of aldehyde through a thermodynamically favorable four-centered transition state (Δ*H*_calc_ = −68 kcal mol^−1^), resulting in the corresponding bis(alkoxo) complex (B_1_). Complex (B_2_) was formed when additional aldehyde molecules were added to the thorium–alkoxide bond. Complex (B_2_) did not add any additional aldehyde molecules, but rather eliminated the first ester (B_4_) in stoichiometric amounts, either by hydride transfer to an incoming aldehyde through a six-centered transition state (B_3_), with the production of the active catalytic complex (B_5_), or by β-H elimination and rapid insertion of an aldehyde to produce the active complex (B_5_). The catalytic cycle started with the insertion of an aldehyde molecule to the thorium–alkoxide bond of complex (B_5_)*via* a four-centered transition state (B_6_), similar to that of complex 10, to produce complex (B_7_), which further performed a hydride transfer to another aldehyde *via* a six-centered transition state (B_8_) as the RDS, thereby regenerating the active catalyst and symmetrical ester as a product (1). A β-hydrogen elimination reaction from (B_7_) could generate the symmetrical ester product (1) as well as a Th–H complex (B_10_), which may react with an aldehyde to generate the active catalytic species (B_5_). The β-hydrogen elimination reaction was found to have a higher enthalpy than the reaction involving a six-centered transition state (+56 and −47 kcal mol^−1^, respectively), which showed that the β-hydrogen elimination pathway was not the primary termination pathway (Scheme 10).

Scheme 10 depicts the two potential reaction pathways available to the aldehyde upon interaction with complex (B_7_). In the first pathway (a), an additional insertion led to the formation of the complex (B_11_), while in the second pathway (b), a hydride transfer produced complex (B_5_) and the ester product (1). The entropy of activation indicated that hydride transfer was more preferred than the additional aldehyde insertion. The Eyring plots and complex 10 were used to ascertain that the Δ*S*^‡^ of activation for all the aldehydes being studied was −65 eu. This result revealed a highly organized transition state with substantial bond-making to compensate for bond-breaking. As the process approaches the transition state, it undergoes a high degree of entropic reorganization. Despite attempts to synthesize complex (B_11_) using independent stoichiometric processes with a bisalkoxo complex and an aldehyde, the desired complex (B_11_) remained elusive. This was surprising from a chemical kinetics standpoint, implying that the aldehyde may be hindered from reaching the metal center due to steric hindrance. In pathway (a), when a four-centered transition state formed, the benzylic carbon should shift to a spatial position close to the oxygen atom bonded to the metal, producing a confined transition state, whereas, in pathway (b), the benzylic carbon should shift to a position proximal to the benzylic hydrogen, resulting in a less hindrance between the incoming aldehyde and the alkoxo group. Comparing the calculated activation energy for pathway a (8.5 kcal mol^−1^) to the measured value for pathway b (3.47 kcal mol^−1^), supported that complex (B_5_) and the dimer were formed during the reaction. The lower values of *E*_a_, and Δ*H*^‡^, as well as the reduced negative value of Δ*S*^‡^ were obtained for complex 10 as compared to complex 8, demonstrating the beneficial effect of incorporation of the bridging ligand resulted in a complex with higher coordinative unsaturation. The use of a constrained-geometry catalyst (CGC) with bridged cyclopentadienyl ligands enhanced the catalytic activity of the corresponding actinide complexes by opening the coordination sphere. The results of this study have opened up a novel pathway for research into the catalytic activity of organoactinide complexes using oxygenated substrates.

Complex 8 promoted “cross-Tishchenko” reaction in which two distinct aldehydes (benzaldehyde and *p*-tolualdehyde) reacted to form four possible esters (Scheme 2). The findings (Table 3) were consistent with the expected influence of aldehyde reactivity on product selectivity. Since benzaldehyde was more active, it predominantly forms a symmetrical ester (1).

**Table 3:** Complex 8 promoted “cross-Tishchenko” reaction between benzaldehyde and *p*-tolualdehyde

S. no.	The ratio of catalyst : benzaldehyde : *p*-tolualdehyde	Yield (%)			
		1	2	3	4
1	1 : 100 : 100	15	4	6	6
2	1 : 100 : 50	25	2	5	5
3	1 : 50 : 100	9	6	6	6

In order to increase the catalyst activity of actinide complexes in the Tishchenko reaction, Karmel and coworkers introduced imidazoline-2-imine (Im^R^N) based actinide catalysts.^99,100^ The imidazolin-2-iminato ligands were potent 2σ,4π-electron donor ligand. The significant delocalization caused by the resonance framework of Im^R^N led to the direct electron donation from the Im^R^N^−^ ligand, and the ligand was characterized as a pseudo-isolobal congener of the Cp moiety.^123–127^

## 3. N-Heterocyclic iminato actinide complexes and neutral lanthanide complexes catalyzed Tishchenko reaction

### 3.1. Imidazolin-2-iminato actinide complexes catalyzed Tishchenko reaction

**Scheme 11:**
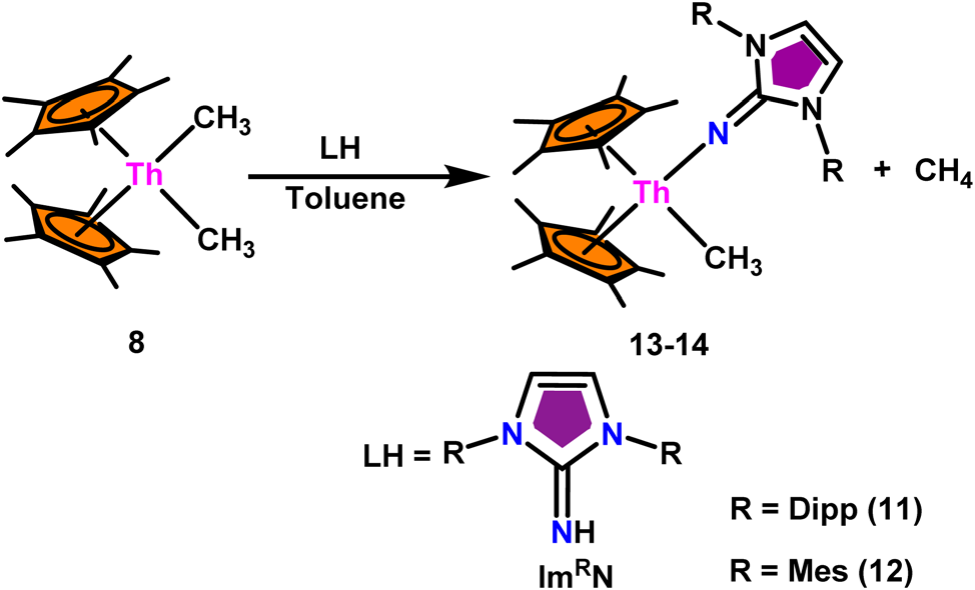
Synthesis of Cp*_2_Th(L)(Me) (13–14).

The mixed pentamethylcyclopentadienyl thorium(iv) imidazolin-2-iminato complexes Th(Im^Dipp^N)(Me)Cp*_2_(13) and Th(Im^Mes^N)(Me)Cp*_2_(14) synthesized from imidazolin-2-imine and toluene solution of Th(CH_3_)_2_Cp*_2_(8) with the immediate evolution of CH_4_ gas (Scheme 11).^100^ The X-ray structure analysis confirmed the central thorium was surrounded by one methyl, one imidazolin-2-iminato moiety, and two Cp* ligands in both complexes 13 and 14, completing the tetrahedral geometry. Complexes 13 and 14 displayed short Th–C_methyl_ bond lengths compared to complex 8. The Th–N_imine_ bond lengths of complexes 13 and 14 were shorter than the Th–N_amido_ bond in Cp*_2_Th(NHR)(Cl) complexes (Scheme 11).

**Scheme 12:**
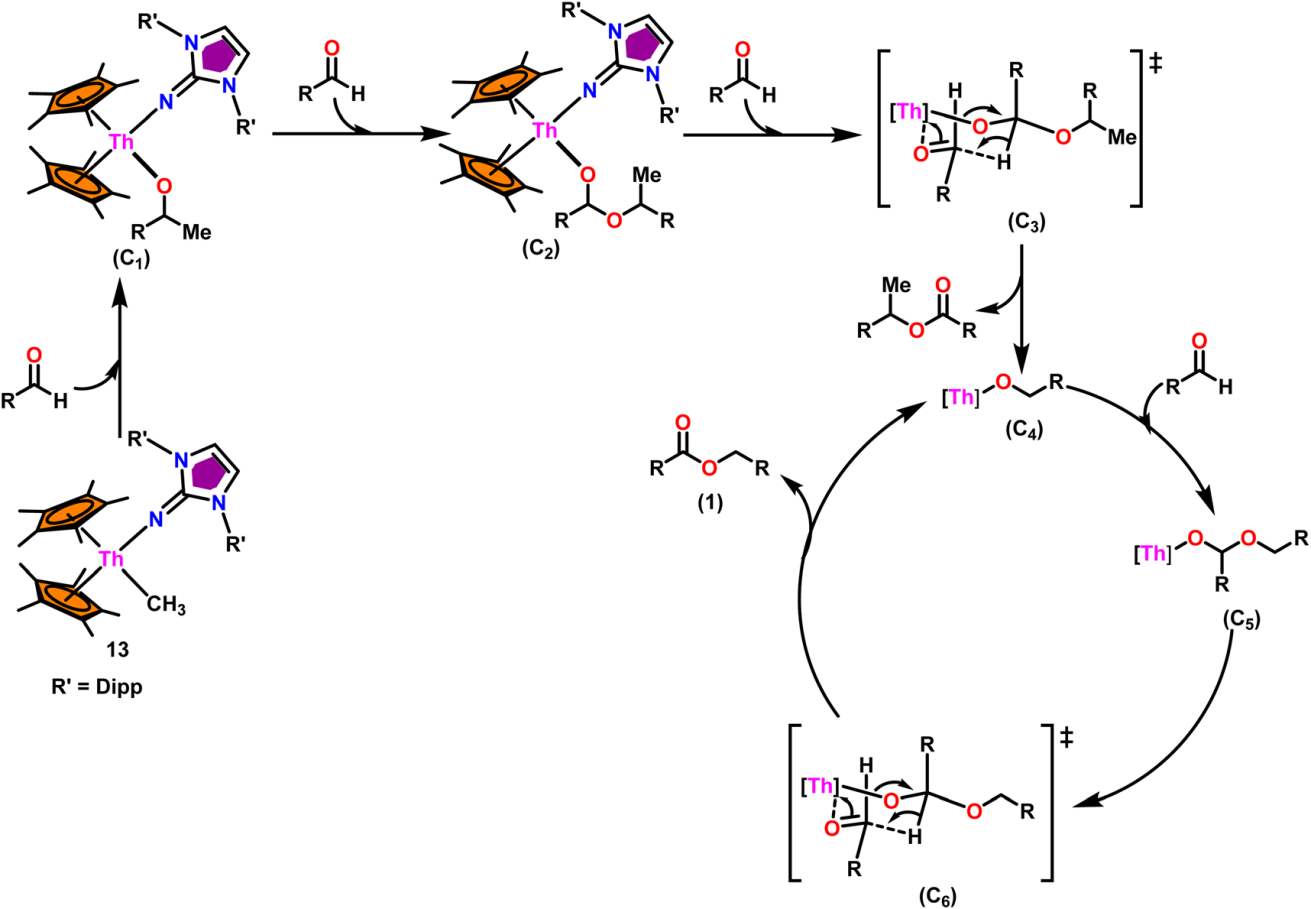
Tishchenko reaction mediated by complex Cp*_2_Th(Im^Dipp^)(Me) (13).

In the catalytic Tishchenko reaction, Menashe *et al.* discovered that enhancing the electron density of a metal complex improved its activity towards aldehydes.^128^ The Tishchenko reaction was performed with various aldehydes including aromatic, heteroaromatic, cyclic, and aliphatic aldehydes. Complex 13 exhibited a slightly greater level of activity compared to complex 14. This can be attributed to the strong electron-donating nature of the imidazolin-2-iminato ligand. It was important to measure the equivalents of precatalyst involved during one catalytic cycle, and the number of active sites present per unit of catalyst for a better understanding of the reaction mechanism. The poisoning experiments carried out using isopropanol suggested that the catalytic activity was reduced to 25% while the catalyst 13 to isopropanol ratio was maintained at 1 : 0.25. However, keeping the catalyst-to-isopropanol ratio to 1 : 0.50, the activity decreased to 50% from the actual value. Similar poisoning experiments with catalyst 8 were found to decrease the activity by 12.5%, keeping the catalyst to isopropanol ratio of 1 : 0.25. When the catalyst 8 to isopropanol ratio was maintained at 1 : 0.50, it led to decreased activity of 1/4^th^ of the initially determined value without isopropanol. So, it was concluded that catalyst 8, containing –CH_3_ groups, was active in the catalytic process. Generally, higher catalytic activity was observed in aldehyde with electron-withdrawing groups which led to higher conversion in a short period of time. For example, benzaldehyde was substituted with *p*-NO_2_, *m*-NO_2_, *p*-CN, and *p*-CF_3_, leading to a higher yield. The presence of the electron-donating groups like methyl or methoxy on the aldehyde made the reaction less efficient, resulting in a lower yield of the desired product. The aromatic and heteroaromatic aldehydes needed a higher reaction time for completion (Table 5). Experimental investigations utilizing precise stoichiometric concentrations of precatalyst 13 and aldehydes provided evidence supporting the coordination insertion mechanism outlined in Scheme 12.^100^ In the first step of the catalytic cycle, the incoming aldehyde was inserted into the Th–C bond, resulting in the thorium alkoxo intermediate (C_1_). After the insertion of a second aldehyde monomer into the Th–O bond, the intermediate (C_2_) was formed. This intermediate, upon hydride transfer with another aldehyde *via* the six-membered transition state (C_3_), led to the catalytically active thorium alkoxo complex (C_4_) as well as 1 equivalent of the methylated ester. Insertion of the incoming aldehyde into the Th–O bond of (C_4_) resulted in the formation of the intermediate (C_5_), which then reacts with another one equivalent of aldehyde *via* a hydride transfer in a six-membered transition state (C_6_) to give the respective ester (1) during regeneration of the catalytically active thorium alkoxo species (C_4_).

This investigation additionally examined the reactivity of thorium(iv) complex 13 in the context of the crossed Tishchenko reaction. When equimolar amounts of two aromatic aldehydes were used in the crossed Tishchenko reaction, the resulting ester mixture showed an almost uniform distribution of all four potential products, indicating a competitive dynamic between the aldehydes (1–4) (Scheme 2). The addition of an excess of different aldehyde (R′CHO) raised the product distribution to the symmetrical ester R′CH_2_OCOR′ (2) (Scheme 2). However, selectivity for the remaining two asymmetrically substituted esters (3–4) remained rather constant, indicating a non-selective reaction pathway. The key outcome of the crossed Tishchenko reaction with a 1 : 1 ratio of RCHO and a heteroatom-substituted R′CHO aldehyde was the symmetrical ester R′CH_2_OCOR′ (2). This suggested that the heteroatom-containing aldehyde had a preference for hydride addition in the reaction mechanism.

**Scheme 13:**
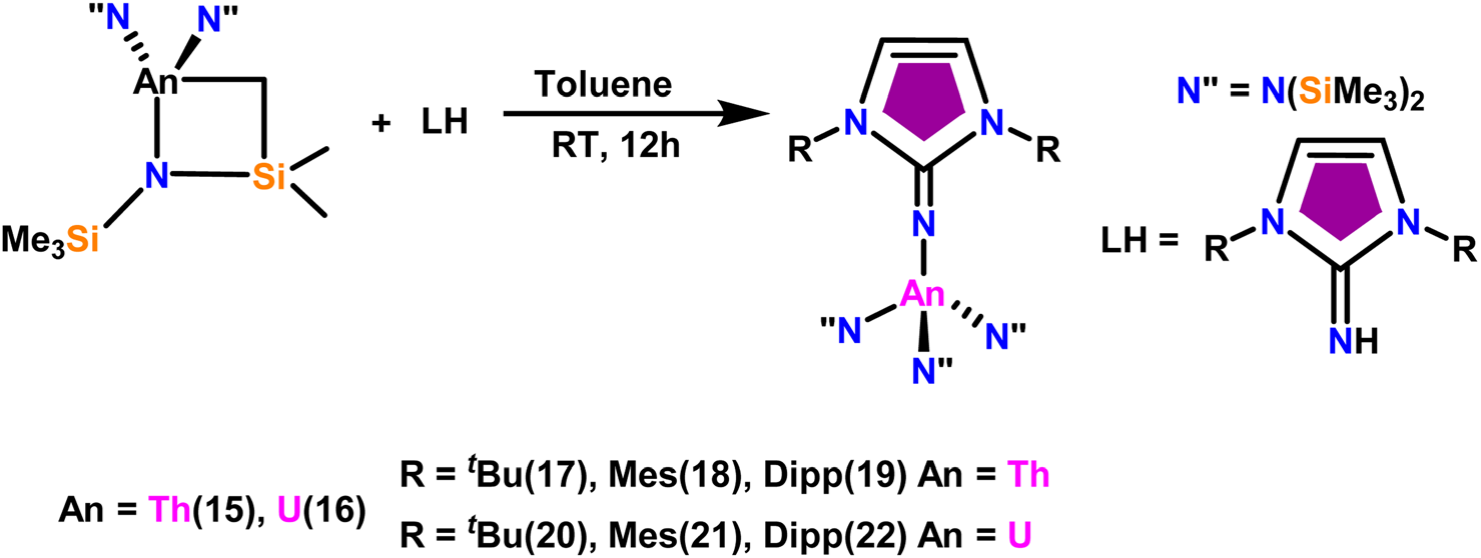
Synthesis of actinide complexes [(Im^Mes^N)Th{N(SiMe_3_)_2_}_3_] (17)–[(Im^Dipp^N)U{(SiMe_3_)_2_}_3_] (22).

**Fig. 1:**
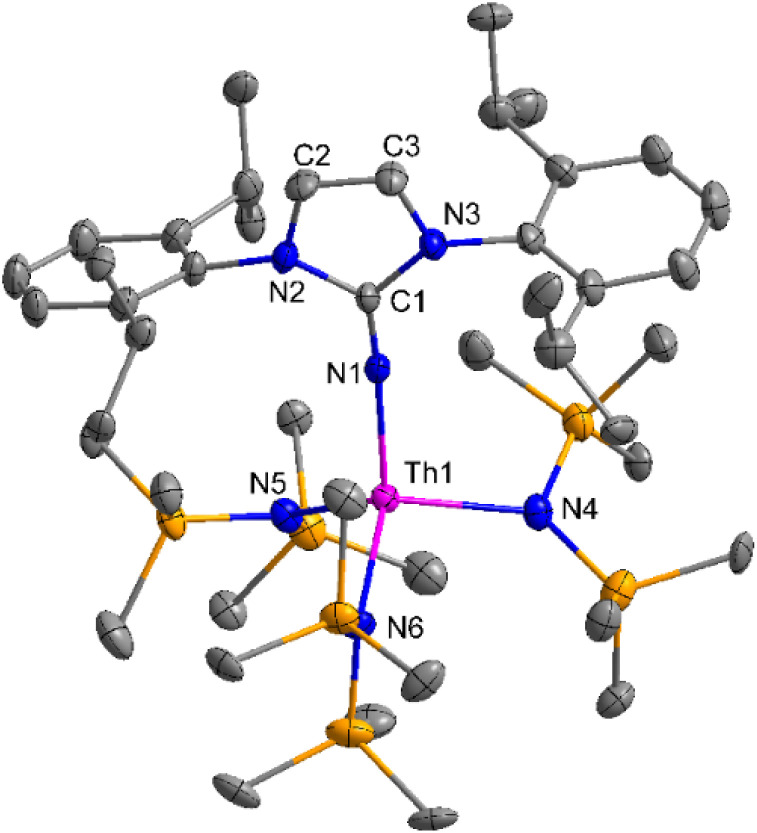
ORTEP diagram of complex 19.

**Scheme 14:**
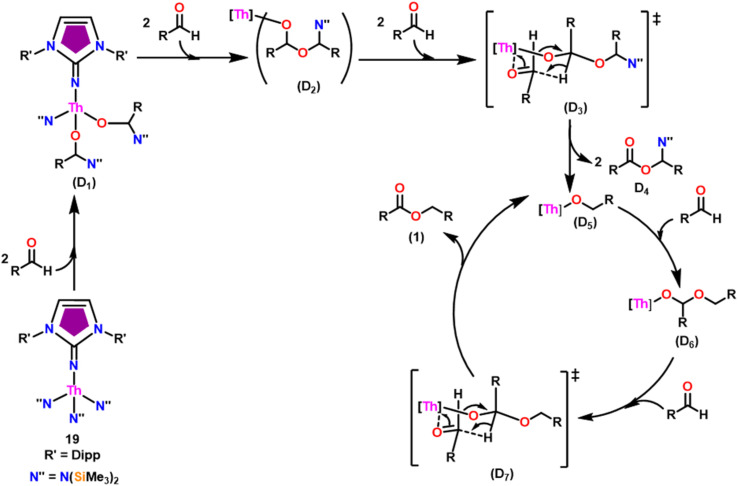
Tishchenko reaction catalyzed by 19.

The imidazolin-2-imines (Im^R^NH, R = *t*-Bu, Mes, Dipp) were protonolysed with actinide metallacycles 15 and 16 to yield the monosubstituted imidazolin-2-iminato actinide(iv) complexes [(Im^*t*Bu^N)Th{N(SiMe_3_)_2_}_3_] (17), [(Im^Mes^N)Th{N(SiMe_3_)_2_}_3_] (18), [(Im^Dipp^N)Th{N(SiMe_3_)_2_}_3_] (19), [(Im^*t*Bu^N)U{N(SiMe_3_)_2_}_3_] (20), [(Im^Mes^N)U{N(SiMe_3_)_2_}_3_] (21) and [(Im^Dipp^N)U{N(SiMe_3_)_2_}_3_] (22) (Scheme 13).^99^ The solid-state structures of these complexes showed marginally deviated tetrahedral geometry. It introduced the concept of the ligand cone angle which was later adapted to other ligand systems by Möhring *et al.*, such as cyclopentadienyl ligands.^129^ This parameter can also be used to describe the steric requirement of imidazolin-2-iminato complexes, with values of 83°, 73°, and 69° for [(Im^*t*Bu^N)U{N(SiMe_3_)_2_}_3_] (20), [(Im^Mes^N)U{N(SiMe_3_)_2_}_3_] (21), and [(Im^Dipp^N)U{N(SiMe_3_)_2_}_3_] (22), respectively. The short An–N_amido_ bond length and large An–N–C_ipso_ bond angle of complexes indicated the higher bond order and strong π-character of the An–N bond. The N–C_ipso_ bond lengths found 1.292(12), 1.308(10), 1.291(14), 1.290(12), 1.313(6), and 1.319(9) Å, for 17–22 respectively (Fig. 1). In early studies, Andrea *et al.* showed the reactivity of Cp*_2_ThMe_2_ towards aromatic aldehydes, which exhibited strong catalytic activity and tolerance for several functional groups.^95^ Karmel and coworkers investigated the aldehyde reactivity of the mono(imidazolin-2-iminato) actinide(iv) complexes 17–22, addressing the fundamental question of whether post-metallocene actinide catalysts exhibit reactivity not only towards aromatic aldehydes but also towards cyclic and branched aliphatic aldehydes. To find a catalyst with the highest catalytic applicability towards the Tishchenko reaction, catalytic studies were performed with benzaldehyde as the model substrate. Thorium complexes were found superior compared to respective uranium analogs. The thorium(iv) complex [(Im^Dipp^N)Th{N(SiMe_3_)_2_}_3_] (19) demonstrated the highest catalytic turnover in the series towards a variety of substrates including aromatic, cyclic, acyclic, polyaromatic, and branched aliphatic aldehydes. The poisoning tests were carried out to assess the percentage of precatalyst that was active in the reaction. Poisoning studies with isopropanol demonstrated that catalyst 19 was actively involved in the catalytic process. Experiments using catalytic amounts of [(Im^Dipp^N)Th{N(SiMe_3_)_2_}] (19) and benzaldehyde showed that two aldehyde units could bind to the Th–N(SiMe_3_)_2_, resulting in twice the amount of N(SiMe_3_)_2_α-substituted ester (D_4_). This product was then characterized using a mixture of ^1^H NMR, ^13^C NMR, ^29^Si NMR, and mass spectroscopy analysis. The space-filling models were used to explore the steric hindrance surrounding the corresponding actinide core, which played a crucial role in catalytic activity. The cavities that were formed by the carbon atoms of the R-substituents of both the imidazolin-2-iminato ligand and the carbon atoms of the bis(trimethylsilyl)amido ligands varied from 3.6 to 4.5 for complexes 17, 18, and 20–22; however, the complex [(Im^Dipp^N)Th{N(SiMe_3_)_2_}_3_] (19) has a larger cavity (5.7), which corresponds to the higher activity. The catalytic cycle started with the coordination of two units of aldehydes to Th-alkoxo species (D_1_) to afford the intermediate species (D_2_).^99^ The intermediate (D_2_) underwent a reaction with another aldehyde, resulting in the formation of the Th–Oxo compound (D_5_)*via* a six-member transition state (D_3_). This process involved the elimination of one equivalent of an ester molecule (D_4_). The desired ester (1) was successfully synthesized by incorporating an aldehyde component into the intermediate (D_6_) through a six-membered transition state (D_7_) and regenerating the catalytically active Th–Oxo species (D_5_). The hydride transfer step was identified as the slowest step in the reaction pathway (Scheme 14).

When complex 19 was employed as a catalyst in the crossed Tishchenko reaction with a 1 : 1 ratio of aromatic/polyaromatic and cyclic/branched aliphatic aldehydes, the resulting products were symmetrical and asymmetrical esters in almost equal quantities (1–4). Surprisingly, employing a 1 : 1 ratio of RCHO and R′CHO, the reaction favored the asymmetric ester (RCH_2_OCOR′) (3) after 2 hours, leaving only a trace of the other symmetric ester (R′CH_2_OCOR′) (2). However, over time (24 hours), the symmetric esters (RCH_2_OCOR (1) and R′CH_2_OCOR′) (2) gain precedence, with traces of another asymmetric ester (R′CH_2_OCOR) (4). The final product distribution was approximately 25% RCH_2_OCOR′ (3), 5% R′CH_2_OCOR (4), 33% RCH_2_OCOR (1), and 35% R′CH_2_OCOR′ (2). This implied that the crucial intermediate (D_5_) (Scheme 14), preferentially interacts with an aliphatic aldehyde. This product can then react with another aromatic aldehyde to complete the catalytic cycle, which is indicated by the initial asymmetric ester formation. As the reaction advanced, increasing amounts of a symmetrical ester revealed competition between the two aldehydes present. To favor the synthesis of the desired, symmetrical ester, the higher proportion of the aromatic aldehyde (RCHO : R′CHO = 20 : 1) was utilized to avoid the formation of an asymmetrical ester. Aliphatic aldehydes, with their superior hydride-donating ability, outcompete electron-withdrawing aromatic aldehydes (better hydride acceptors) for interaction with the catalyst. This control over the reaction pathway leads to the selective formation of the symmetric ester. This suggested that the rate-determining step (RDS) in the catalytic cycle was hydrogen transfer. When the amount of aliphatic aldehyde (R′CHO) decreases (over 95% of the target product formed), the excess aromatic aldehyde (RCHO), acts as a slower hydride acceptor, which gradually forms the symmetrical ester, reaching complete conversion after 24 hours (Table 4).^99^

**Table 4:** Crossed Tishchenko reaction catalyzed by complex 19^a^

S. no.	RCHO	R′CHO	Yield (%)			
			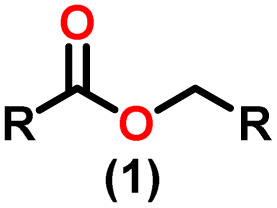	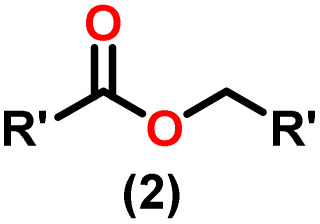	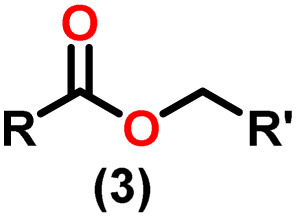	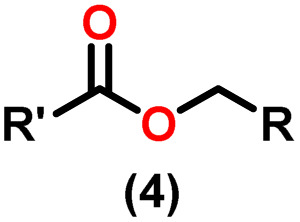
1	Ph	C_6_H_11_	—	—	92	8
2	Ph	C_5_H_9_	—	12	84	—
3	Ph	Isopropyl	—	20	80	—
4	1-Naphthyl	C_6_H_11_	5	—	100	—
5	2-Naphthyl	C_6_H_11_	5	—	88	12

a Reaction conditions: 4.48 μmol of catalyst 19, cat/RCHO/R′CHO = 1/200/50, 750 μL of C_6_D_6_; RT, yield was determined by ^1^H NMR spectroscopy.

**Table 5:** Dimerization of aldehydes by N-heterocyclic imine-based catalyst

S. no.	Catalyst	RCHO [% yield]
1	Cp*_2_Th(Im^Dipp^N)(Me) (13)	Ph-[60], 4-NO_2_Ph-[95], 1-naphthyl-[77], 2-naphthyl-[45], 2-pyridyl-[83], 2-furyl-[43], 2-thiophen-[22], cyclohexyl-[100], cyclopentyl-[100], iso-propyl-[100]
2	[(Im^Dipp^N)Th{N(SiMe_3_)_2_}_3_] (19)	Ph-[60], 1-naphthyl-[77], 2-naphthyl-[45], 4-NO_2_Ph-[95], cyclohexyl-[100], cyclopentyl-[100], isopropyl-[100], *o*-Ph(CHO)_2_-[100]

### 3.2. Benzimidazolin-2-iminato actinide complex based tandem proton-transfer esterification (TPTE)

**Scheme 15:**
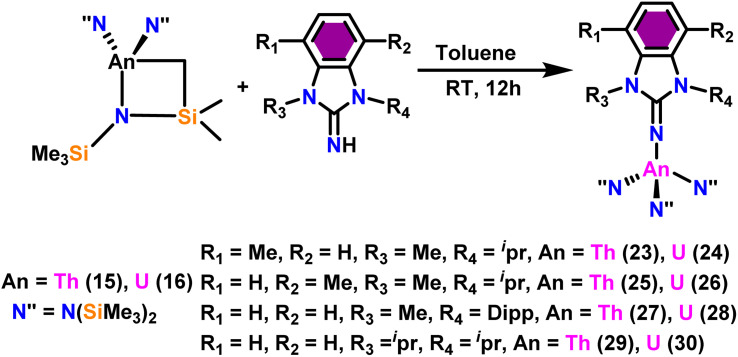
Synthesis of benzimidazolin-2-iminato actinide complexes 23–30.

**Scheme 16:**
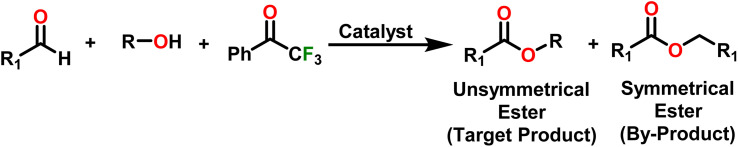
TPTE of alcohols and aldehydes.

**Fig. 2:**
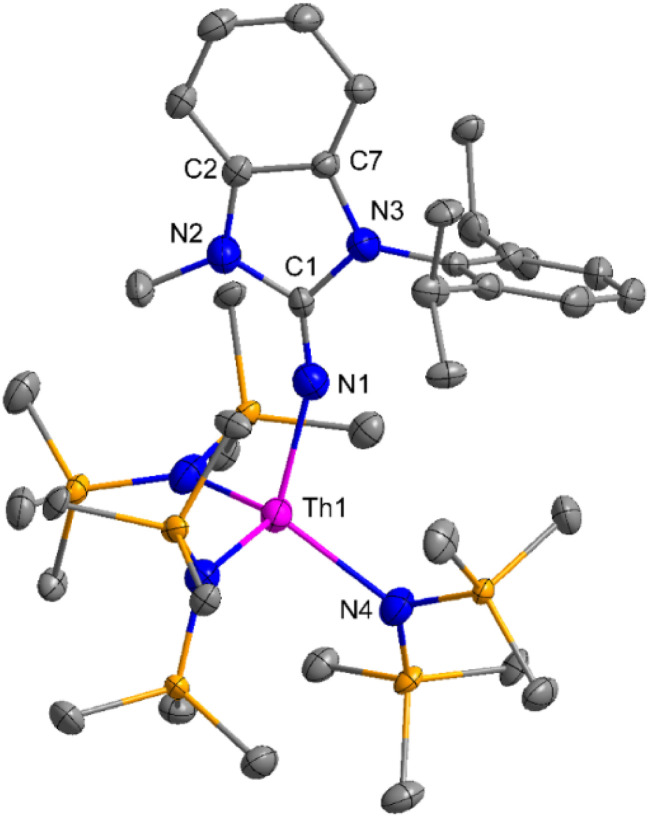
ORTEP diagram of complex 27.

Liu and co-workers have developed a unique series of ligand systems through alterations to the imidazolin-2-imine backbone, intending to impact the catalytic effectiveness of actinide complexes.^53,101^ The synthesized ligands were used to form a new class of actinide complexes by treating with actinide metallacycles [(Me_3_Si)_2_N]_2_An-[*κ*^2^(*N,C*)-CH_2_Si(CH_3_)_2_N(SiMe_3_)] (An = Th (15) and U (16)) to generate a series of actinide complexes (Scheme 15). Solid-state studies of all complexes revealed identical isomorphous pseudo-tetrahedral geometries and bonding characteristics to the corresponding imidazolin-2-iminato actinide complexes (23–30).^53,99^ Tandem proton-transfer esterification (TPTE) represented a novel approach for the synthesis of asymmetric esters by coupling aldehydes and alcohols (Scheme 16). The benzimidazolin-2-iminato actinide(iv) complexes (23–30) were utilized for the Tishchenko reaction. The reaction conditions were optimized by carrying out the reactions between PhCHO/CH_3_OH/27. The threefold increase in the molar ratio of the PhCHO/CH_3_OH from 1 : 1 to 3 : 1, enhanced the yields of methyl benzoate, indicating the advantageous presence of aldehyde in the reaction mixtures. While increasing the methanol amount in the mixture from 1/1 to 1/3, a reduced yield of the asymmetric ester demonstrated the negative influence of alcohol present in the reaction mixture. In general, thorium complexes exhibited higher activity compared to uranium analogs. In the tandem proton transfer esterification reaction, a special ketone called α,α,α-trifluoroacetophenone (TFMAP) used as sacrificial ketone to avoid unwanted symmetrical products (Fig. 2 and Table 6).

**Table 6:** Proton transfer esterification in the presence of the sacrificial ketone TFMAP by catalyst 27^a^

S. no.	[R_1_CHO]	[ROH]	PhCOCF_3_/MeOH	Yield of R_1_COOR^b^ (%)
1	PhCHO	MeOH	1/1	71
			2/1	68
2	4-MePhCHO	MeOH	1/1	47
			2/1	28
3	2-Naphthyldehyde	MeOH	1/1	65
			2/1	43
4	PhCHO	MeOH	1/1	20

a Self-dimerization of aldehyde produces an unavoidable small number of symmetrical esters (1) along with the desired unsymmetrical ester (E_4_) (Scheme 16).^53,101^

b Yield was determined by ^1^H NMR spectroscopy from the crude reaction mixture.

**Scheme 17:**
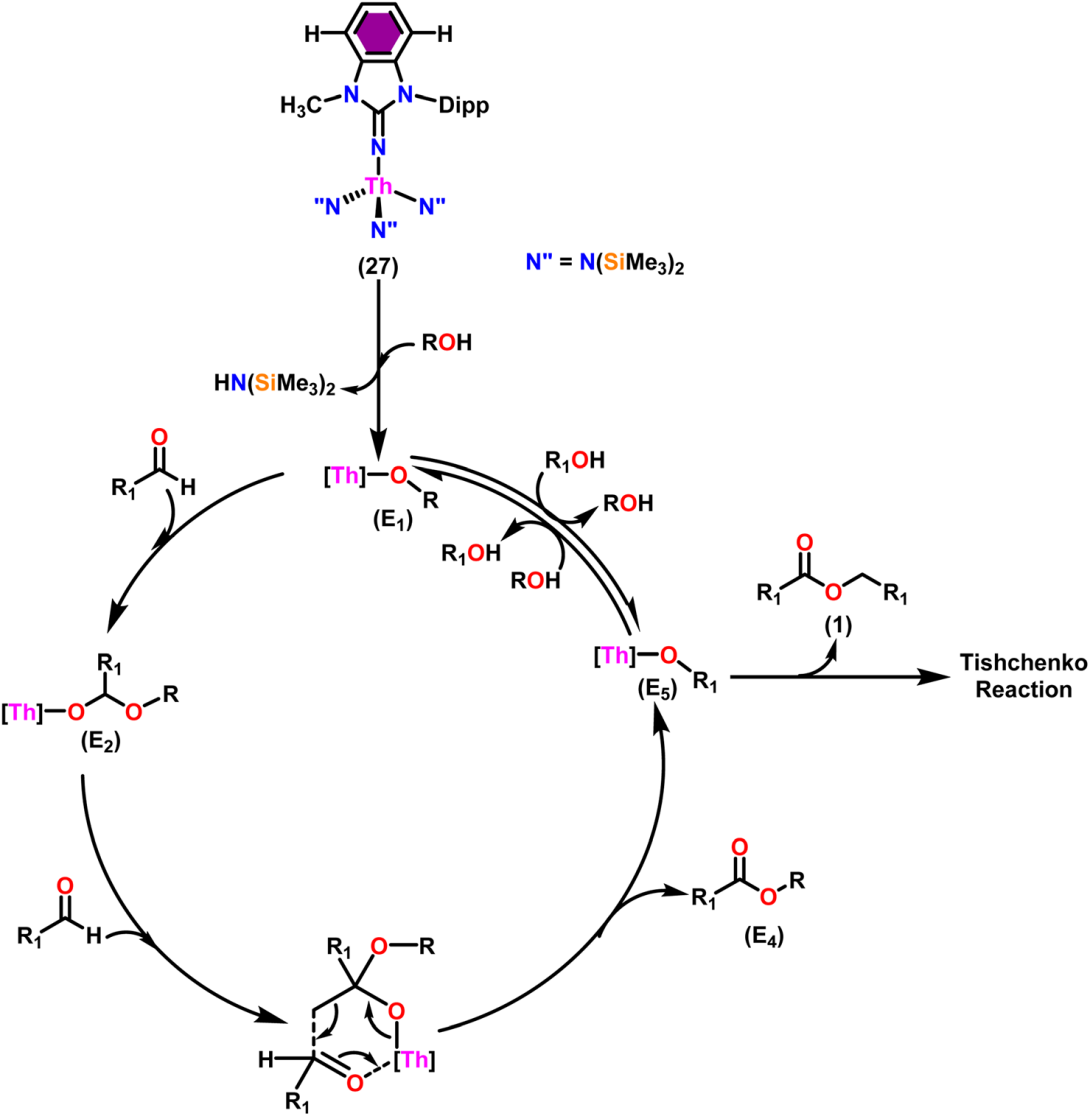
The mechanism for tandem proton-transfer esterification (TPTE) of alcohols and aldehyde.

Taking into consideration the findings, a viable mechanism was suggested.^53,101^ The catalytic cycle commenced with the reaction of complex 27 and alcohol, leading to the formation of actinide alkoxide species (E_1_) (Scheme 17). The catalytically active species (E_1_) was inserted into the carbonyl group of incoming aldehydes to form an intermediate (E_2_). The intermediate (E_2_) coupled with another approaching aldehyde *via* a six-member transition state (E_3_) to yield actinide alkoxide species (E_5_) and the target asymmetric ester (E_4_). Proton transfer takes place between the (E_5_) and excess alcohol, which regenerates the active catalyst (E_1_).

Substrates having electron-withdrawing groups were more active than those having electron-donating substituents. The inclusion of potent electron-donating groups like 4-MeO–, 4-Me_2_N– significantly reduced the reactivity of the active species. The steric nature of the substituents had a notable impact on the reaction. As the hindrance increased, the yield decreased because bulky aldehydes or alcohols were unable to easily get to the thorium center. The N-heterocyclic imine's central structure can be altered by varying the size of the ring. Through the incorporation of the perimidin-2-iminato (Pr^R^N^−^) moiety, a remarkable achievement was made in expanding the ring and modifying the backbone on a single platform.^102^

### 3.3. Perimidine scaffold and seven-membered N-heterocyclic iminato actinide complexes catalyzed Tishchenko reaction

**Scheme 18:**
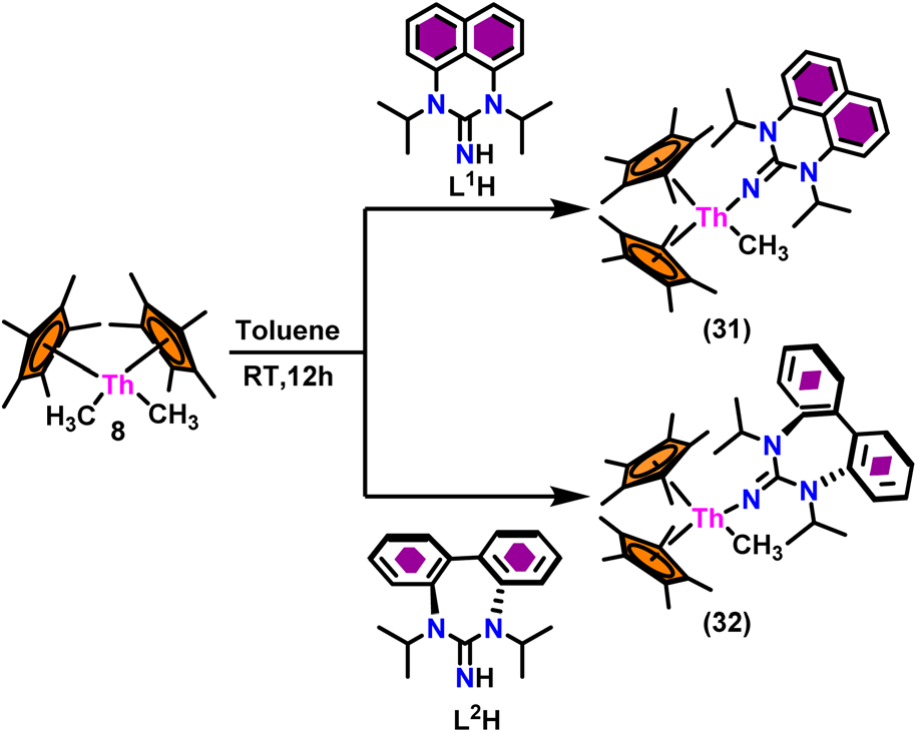
Synthesis of Cp*_2_Th(L^1^)(Me) (31) and Cp*_2_Th(L^2^)(Me) (32).

The flat structure of the five and six-membered N-heterocyclic iminato auxiliary ligands imposes limitations on the spatial arrangement of the wingtip. The incorporation of a six-membered ring instead of a five-membered heterocycle moiety, as detailed in the study by Ghatak *et al.*, led to the development of the Perimidine scaffold. This modification resulted in a notable increase in the activity of organoactinide complexes.^102^ A novel N-heterocyclic iminato core architecture featuring a seven-membered ring was designed to address the rigidity challenges associated with planar heterocyclic frameworks. This innovative design incorporated a torsional twist mechanism to alleviate ring strain and enhance functionality. This study showed the simple synthesis of two Th(iv) complexes, one with a six-membered N-heterocyclic iminato framework and the other with a new seven-membered ring ligand. The impact of ring size on the catalytic activity of an organoactinide complex was investigated in this study. The actinide complexes 31 and 32 were synthesized in toluene using a one-pot reaction between complex 8 with the respective N-heterocyclic imine ligand, which resulted in the immediate evolution of methane (Scheme 18).

**Fig. 3:**
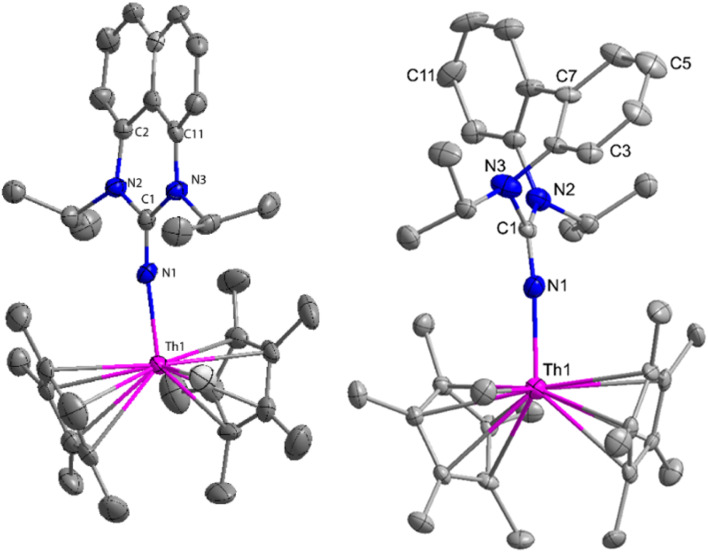
ORTEP diagram of complex 31 and 32.

The X-ray structure study of complex 31 revealed that the distance between the Th–N1_imine_ bond (2.225 Å) and other imidazolin-2-iminato thorium structures was comparable, showing that the Th–N bond has a high bond order (Fig. 3). The Th–Cp* centroid distances were 2.57 Å and 2.56 Å, respectively, while the Th–CH_3_ = 2.488 Å, all of which were shorter than those observed for Cp*_2_Th(CH_3_)_2_. The crystal structure of the free ligand L^2^H revealed the axial symmetry resulting from the twisting of the seven-membered N-heterocyclic imine rings (42.2° torsional angles between two phenyl rings). The distances between the Th–N bond and the Th–Cp*centroid for complex 32 were 2.21, 2.55, and 2.58 Å respectively (Fig. 3). The equivalent bond distances observed in complexes 31 and 32 indicated that these complexes possess comparable electrical properties. Complex 31 and 32 were employed as a precatalyst to efficiently transform aldehydes into their respective esters *via* the Tishchenko reaction, achieving excellent yields even at ambient temperature. The broad range of substrates includes aromatic, heteroaromatic, aliphatic, and cyclic aldehydes used to dimerize aldehydes. In general, the activity of complex 31 was either identical to or slightly better than that of complex 32. Aromatic aldehydes containing electron-donating groups react faster than those containing electron-withdrawing groups. The most active substrates were found to be cyclic or branched aliphatic aldehydes. The yield of the product depended on the position of the substituent on the phenyl ring. The substrates that interact with the highly electrophilic thorium catalyst exhibited a decrease in reactivity, restricting the coupling of the aldehyde and the metal-alkoxo moiety. When complex 31 was employed to perform the cross-Tishchenko reaction of benzaldehyde and 1-naphthaldehyde, all four potential ester products formed as expected (Table 7). However, the reaction initially preferred the formation of an unsymmetrical ester (3) over symmetrical ones (1 and 2). Over time, the reaction shifted towards symmetrical esters, with the other unsymmetrical ester (4) becoming nearly undetectable. Chemoselective ester (3) formation was achieved by using a ratio of 1.5 : 1 of the aldehyde (RCHO : R′CHO) in the reaction mixture. A cross-Tishchenko reaction of benzaldehyde and pyridine-2-carbaldehyde in a 1 : 1 ratio yielded four different types of esters, with ester (3) dominating. This selectivity towards ester (3) was attributed to the increased benzaldehyde-to-pyridine-2-carbaldehyde ratio (1.5 : 1). The selectivity of this reaction was likely attributed to the hydride acceptor and donor characteristics of the aldehydes. The cross-Tishchenko reaction of benzaldehyde with aliphatic aldehydes (cyclohexanecarboxaldehyde, isobutyraldehyde) produced a negligible ester (3). Whereas, aliphatic aldehydes preferentially donate hydride, favoring esters (2) and (4). Poisoning experiments were also performed using the equimolar amount of isopropanol and a catalyst 31 demonstrated that increasing the proportion of isopropanol from 0.25 to 0.5, showed a decline in the catalytic activity from 25% to 50%, suggesting that all methyl groups were active in the catalytic cycle. Kinetic studies of the initial rates of the reaction with complex 31 and 32 determined that the reaction follows a first-order dependence for aldehyde and complex. Therefore, an apparent rate law for the dimerization of aldehyde promoted by precatalysts 31 and 32 can be expressed as indicated in eqn (3).

3 d*p*/d*t* = *K*_obs_ × [31 or 32]^1^ × [PhCHO]^1^

**Table 7:** Crossed Tishchenko reaction catalyzed by complex 31^a^^,^^e^

S. no.	RCHO	R′CHO	Yield (%)			
			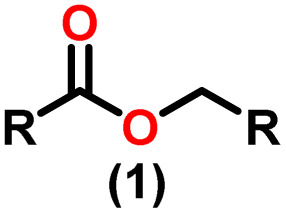	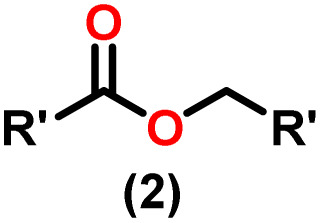	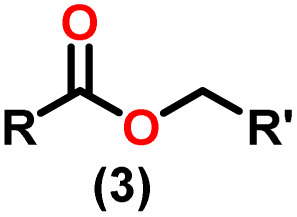	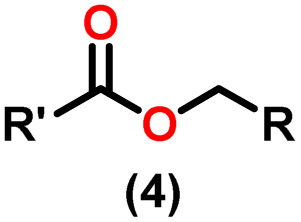
1^b^	Ph	1-Naphthyl	17	16	62	4
2^c^	Ph	1-Naphthyl	—	—	95	—
3^b^	Ph	2-Pyridyl	3	6	80	10
4^c^	Ph	2-Pyridyl	—	—	100	—
5^c^	Ph	Cyclohexyl	27	46	—	26
6^d^	Ph	Cyclohexyl	20	35	—	40
7^b^	Ph	Isopropyl	18	55	6	25
8^d^	Ph	Isopropyl	46	28	11	15
9^c^	Ph	Isopropyl		77	—	23

a Reaction conditions: catalyst 31/RCHO = 1 : 100, RT, 1.5 h, 0.5 mL of C_6_D_6_.

b RCHO : R′CHO = 1 : 1.

c RCHO : R′CHO = 1 : 1.5.

d RCHO : R′CHO = 1.5 : 1.

e Analysis done by ^1^H spectroscopy.

A primary kinetic isotopic effect of 2.7 was measured when the reaction was studied with α-deuterated benzaldehyde. This study revealed that hydride transfer was involved in a rate-determining step. According to thermodynamic studies, the energy of activation (*E*_a_), enthalpy of activation (Δ*H*^‡^), and entropy of activation (Δ*S*^‡^) were 3.48 kcal mol^−1^, 2.63 kcal mol^−1^, and −68.4 eu, respectively. The significantly negative value of entropy at the RDS indicated a well-ordered transition state.

**Scheme 19:**
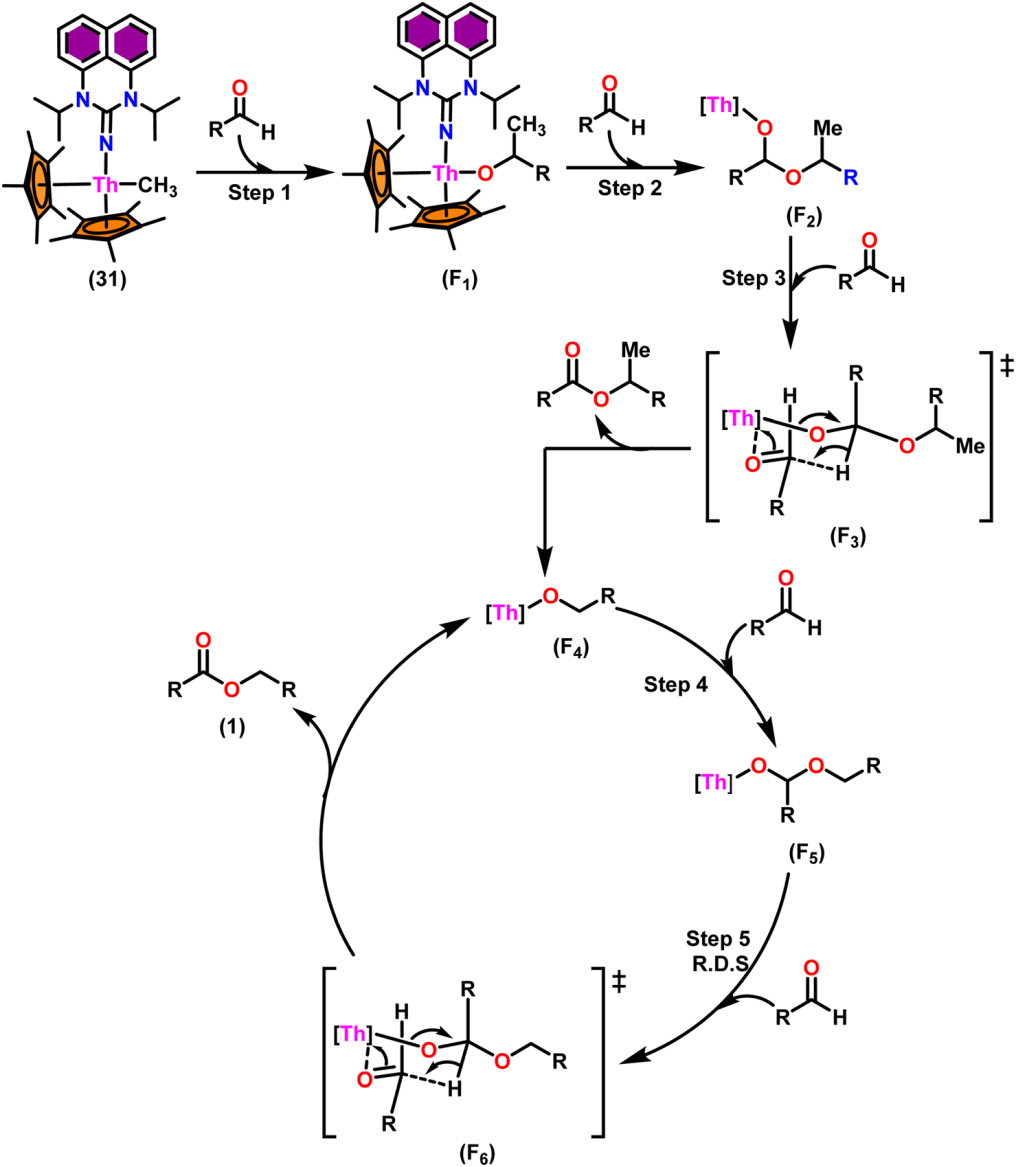
Proposed mechanism for the Tishchenko reaction mediated by complex 31.

Scheme 19 depicts a plausible mechanism for the dimerization of aldehydes by complex 31.^102^ In the first step of the reaction, an aldehyde was rapidly inserted into the Th–CH_3_ bond using a four-centered transition state to form the thorium alkoxo intermediate (F_1_). When a second aldehyde was introduced into the Th–O bond, an intermediate (F_2_) was formed. Subsequent hydride transfer to an incoming aldehyde *via* a chair-like six-membered transition state (F_3_) produced the catalytically active intermediate (F_4_) and one equivalent of the methylated ester. The addition of an aldehyde to the Th–O bond of the intermediate (F_4_) led to the formation of the intermediate complex (F_5_), which after undergoing a hydride transfer reaction (step 5, RDS) with an additional aldehyde, brought the catalytic cycle to an end to give the product (1) and regenerated the active complex (F_4_).

### 3.4. Ethyllanthanoid iodide complex catalyzed Tishchenko reaction

Yokoo *et al.* discovered the ethyl lanthanoid iodide complexes (EtLnI) [Ln = Pr (33a), Nd (33b), Sm (33c)] act as catalytic precursors for the Tishchenko reaction of different types of aldehydes.^103,130–135^ The catalysts 33a–33c were synthesized from the reaction between lanthanide metal (Ln = Pr, Nd, Sm) and ethyl iodide. In the presence of catalysts 33a–33c, arylic and aliphatic aldehydes were shown to undergo Tishchenko reactions with identical catalytic activity. It has been found that Pr, Nd, and Sm undergo Tishchenko reactions, while the more stable divalent lanthanide species, like Yb and Eu, only show Grignard reactions.^131–135^ Even though divalent lanthanide species like Yb and Eu demonstrated catalytic activity for the Tishchenko reaction, the yield was lower. Despite the prevalence of aluminum alkoxides,^28,136,137^ as catalysts for the Tishchenko reaction, the lanthanide-based investigation revealed the potential of compounds 33a–33c as alternative catalytic precursors for the Tishchenko reaction.

**Scheme 20:**
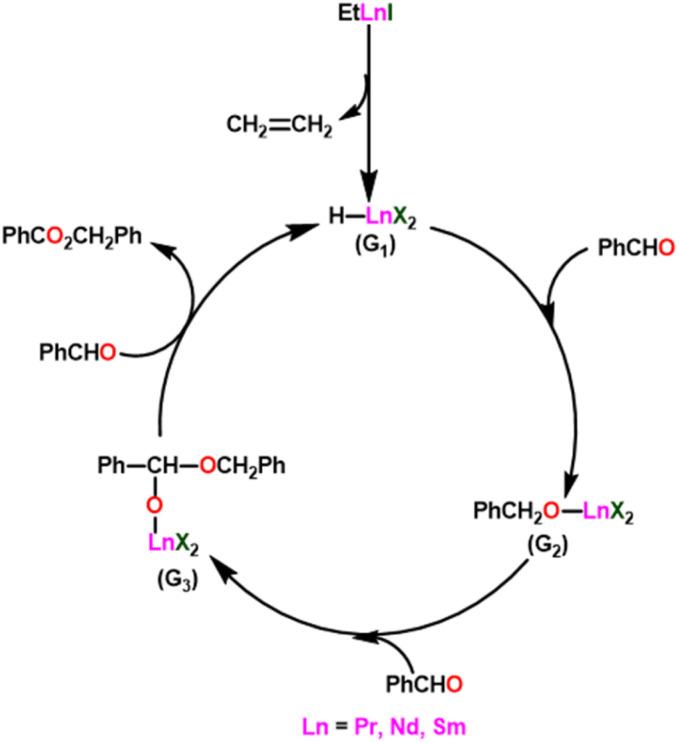
The plausible mechanism of 33 catalyzed Tishchenko reaction.

The mechanism was shown in Scheme 20, which was similar to that of the reaction with the aluminum alkoxides.^103^ The first step was the elimination of the ethylene molecule from 33 to generate the active catalyst (G_1_). The formation of the lanthanide–alkoxide complex PhCH_2_O–LnX_2_(G_2_), was achieved by β-hydride elimination, through an additional reaction between (G_1_) and benzaldehyde. After which the second molecule of benzaldehyde was added to afford an intermediate (G_3_). Again, the addition of benzaldehyde to intermediate (G_3_) afforded the corresponding ester and (G_1_). Despite the tungsten-promoted Tishchenko reaction was already reported, this was the first example of a lanthanide-catalyzed Tishchenko condensation of aldehydes.

### 3.5. Lanthanocene complex catalyzed Tishchenko reaction

**Scheme 21:**
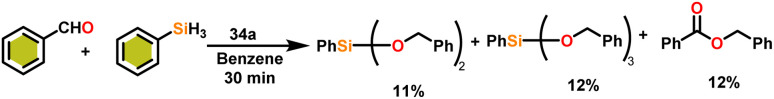
Reaction of the aldehyde with phenylsilane.

Onozawa and coworkers reported that lanthanocene complexes, Cp*_2_LnCH(SiMe_3_)_2_(34) [Ln = Nd (34a), La (34b)] were found to be an efficient catalyst for Tishchenko reactions.^104^ The Tishchenko product was unexpectedly produced in a substantial amount during the hydrosilylation process of benzaldehyde with catalyst 34a (Scheme 21).^138^ It has been observed that the complex 34b was more active than 34a.

**Scheme 22:**
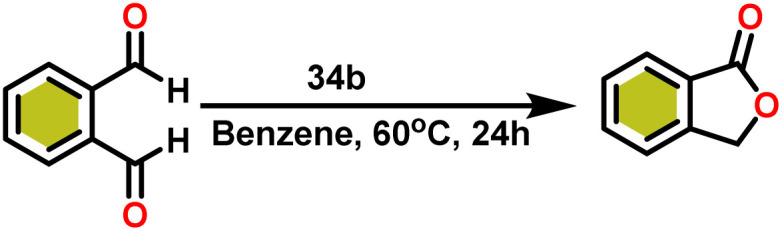
Intramolecular Tishchenko reaction of *o*-phthalaldehyde catalyzed by 34b.

**Scheme 23:**
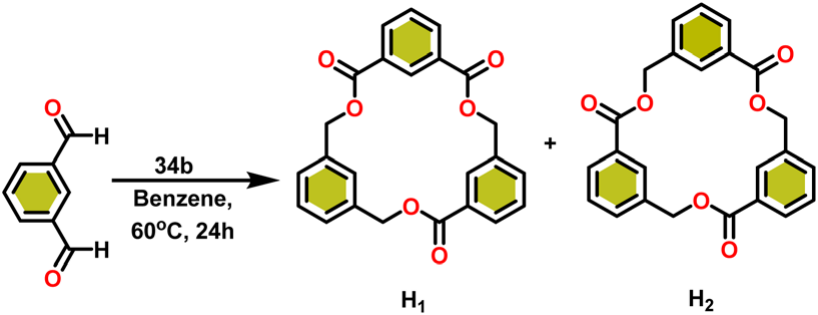
Tishchenko reaction of isophthalaldehyde catalyzed by 34b.

Ishii *et al.* have found that 5 (ref. 48) acts as an active catalyst for the transformation of benzaldehyde into benzyl benzoate. In comparison to transition metal catalysts like 5 and 6,^49^ the activity of the lanthanocene complexes (34a–34b) was higher. Furfural dimerization was extremely difficult in the presence of 34 or 7 catalysts.^50^ The use of 7 in combination with crown ethers as a catalyst resulted in the production of esters with good yield.^50,136,139^ Only aliphatic aldehydes with branching α-carbon (pivalaldehyde and cyclohexanecarboxaldehyde) produced the corresponding dimers in good yield whereas the aldehydes without α-branching afforded dehydrated trimers as the major product. The catalyst 34b was more active than commercial La(OCHMe_2_)_3_(35),^104^ which was very sensitive to the structure of the substrate. It has been noted that the esters were formed in good yield when *p*-substituted benzaldehydes were used as a substrate. The activity of aromatic aldehydes was improved by incorporating electron-withdrawing substituents in the *para* position. The yield trend of *p*-substituted benzaldehydes was expressed as follows: MeO < Me < Cl < NC. The Tishchenko reaction of dialdehyde was studied using *o*-phthalaldehyde. The reaction of *o*-phthalaldehyde in the presence of catalyst 34a afforded phthalide in good yield (Scheme 22). The GC-MS analysis revealed that the intermolecular dimerization product was not formed during this reaction. The presence of catalyst 34b led to the formation of oligomers (*M*_w_ = 300) and polymers (*M*_w_ = 6300) as a result of the reaction of isophthalaldehyde, with the latter being the predominant product (Scheme 23). When the reaction mixture was heated, the molecular weight of the polymer increased but the quantity of the polymer was decreased. After prolonged heating, the oligomers were formed as a major product. The transformation of the polymer into a thermodynamically stable oligoester indicated the occurrence of an ester exchange reaction in the presence of 34b.

**Scheme 24:**
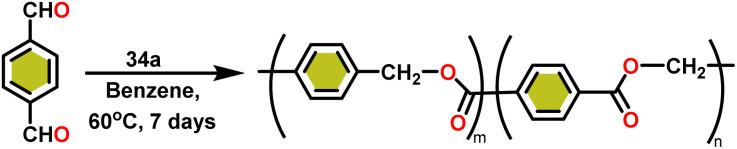
Tishchenko reaction of terephthalaldehyde catalyzed by 34a.

The formed oligomers contain two isomers (H_1_) and (H_2_). The structures of oligomers were possibly to be cyclic triesters of isophthalaldehyde. This was confirmed by the absence of an aldehydic proton signal in ^1^H NMR analysis. The three (1 : 1 : 1) methylene signals and three (1 : 1 : 1) carbonyl signals of the ^13^C NMR spectrum show that the main isomer (H_1_) has an irregular structure. The reaction of terephthalaldehyde in the presence of catalyst 34a afforded the corresponding polymer in good yield (Scheme 24). In this reaction, there was no intramolecular dimerization and oligomerization (like *ortho* and *meta* isomer respectively) observed. It was found that the molecular weight of the polymer increased as the reaction time increased. The polymer's irregular structure resulted from the presence of two signals in the ^1^H NMR and three methylene signals in the ^13^C NMR spectrum, indicating its complex composition.

**Scheme 25:**
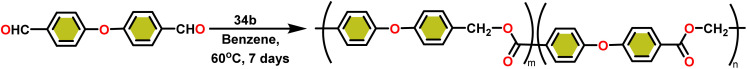
Polymerization reaction of di(4-formylphenyl)ether.

**Scheme 26:**
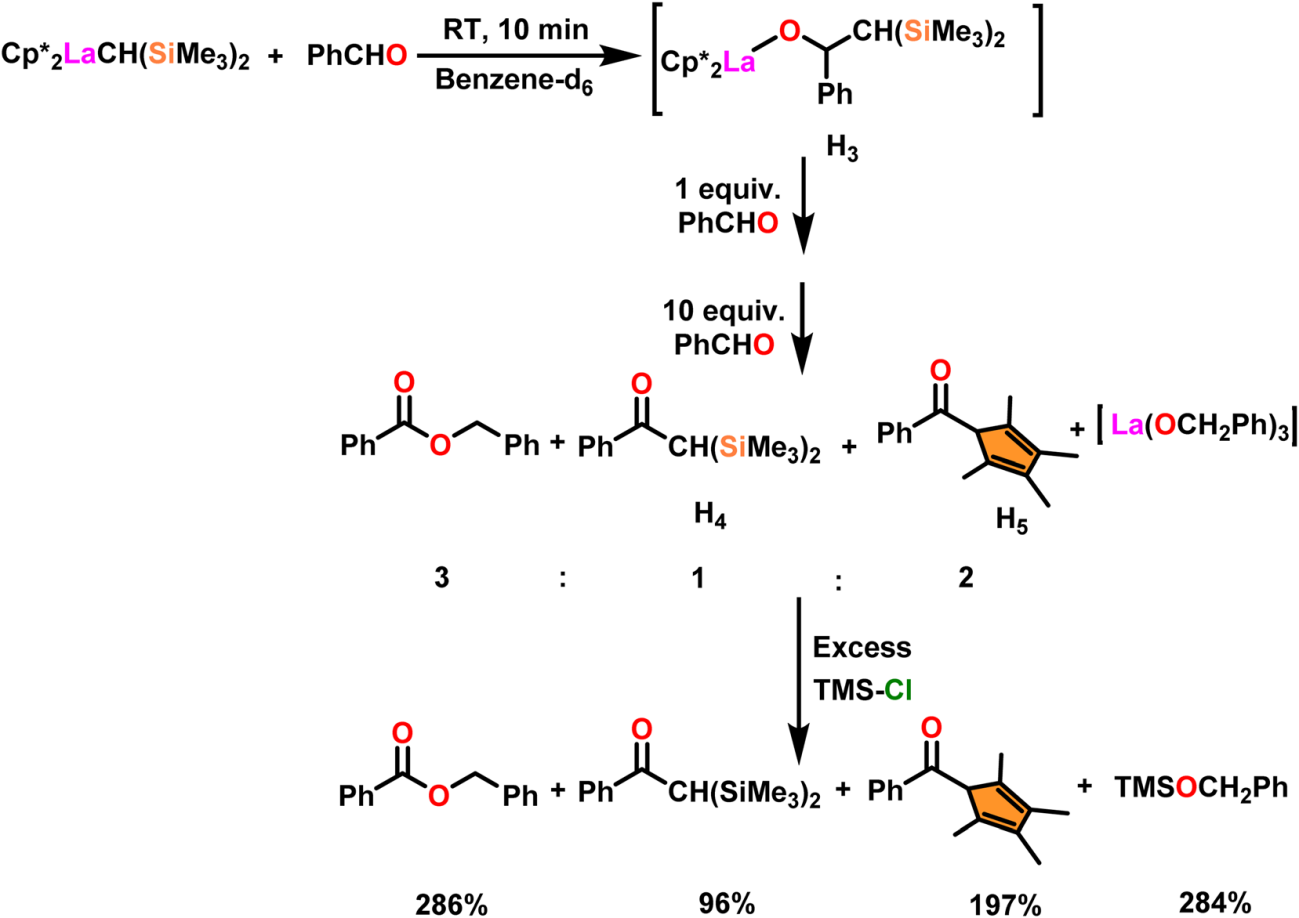
Reaction mechanism of Tishchenko reaction in the presence of catalyst 34b.

The process involving the polymerization of di(4-formylphenyl)ether afforded the corresponding polymer with high molecular weight (Scheme 25). The mechanism of the Tishchenko reaction was briefly explained in Scheme 26.^104^ Complex 34b reacted with benzaldehyde to afford alkoxo complex (H_3_). The formation of (H_3_) was confirmed by new mutually coupled doublets at *δ* 0.94 ppm (methine proton attached to two silicon atoms) and at *δ* 5.33 ppm (benzylic proton) in the ^1^H NMR spectrum. The complex (H_3_) was treated with an excess of benzaldehyde to produce benzyl benzoate, ketone (H_4_), and pentamethylcyclopetadienyl phenyl ketone (H_5_). In the ^1^H NMR spectrum, the signal at *δ* 6.9 ppm (broad) indicated the formation of PhCH_2_OLa species with corresponding ketones. The formed PhCH_2_OLa species was confirmed by the addition of a large amount of chlorotrimethyl silane to the former mixture which resulted in the production of trimethylsilyl benzyl ether. As a result, benzyloxy-La was identified as the active species during the Tishchenko reaction.

**Scheme 27:**
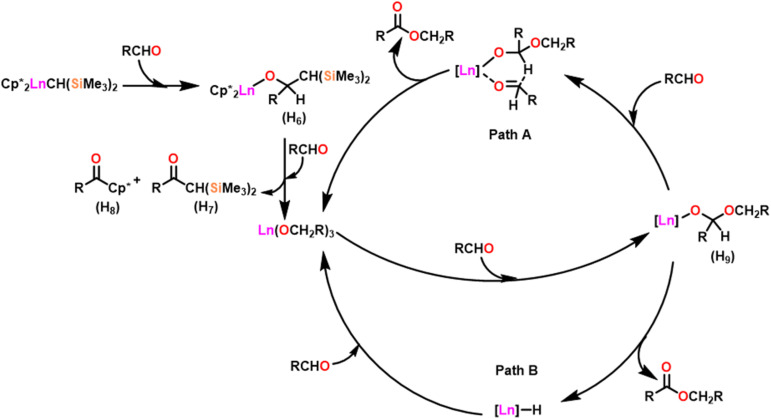
Catalytic cycle for the Tishchenko reaction.

Scheme 27 depicts the catalytic cycle and its mechanism.^104^ The first step in the catalytic cycle involved the aldehyde insertion into the Ln–OR bond, resulting in the formation of hemiacetal-Ln species (H_9_). After the formation of (H_9_), there were two different pathways. In pathway A, an aldehyde was coordinated to a metal ion (H_9_), and the hydride subsequently migrated through a six-membered transition state and led to the formation of a product. Path B proceeds with direct β-hydride elimination from (H_9_) forming Ln–H species followed by aldehyde insertion.

### 3.6. Lanthanide amides catalyzed Tishchenko reaction

**Scheme 28:**
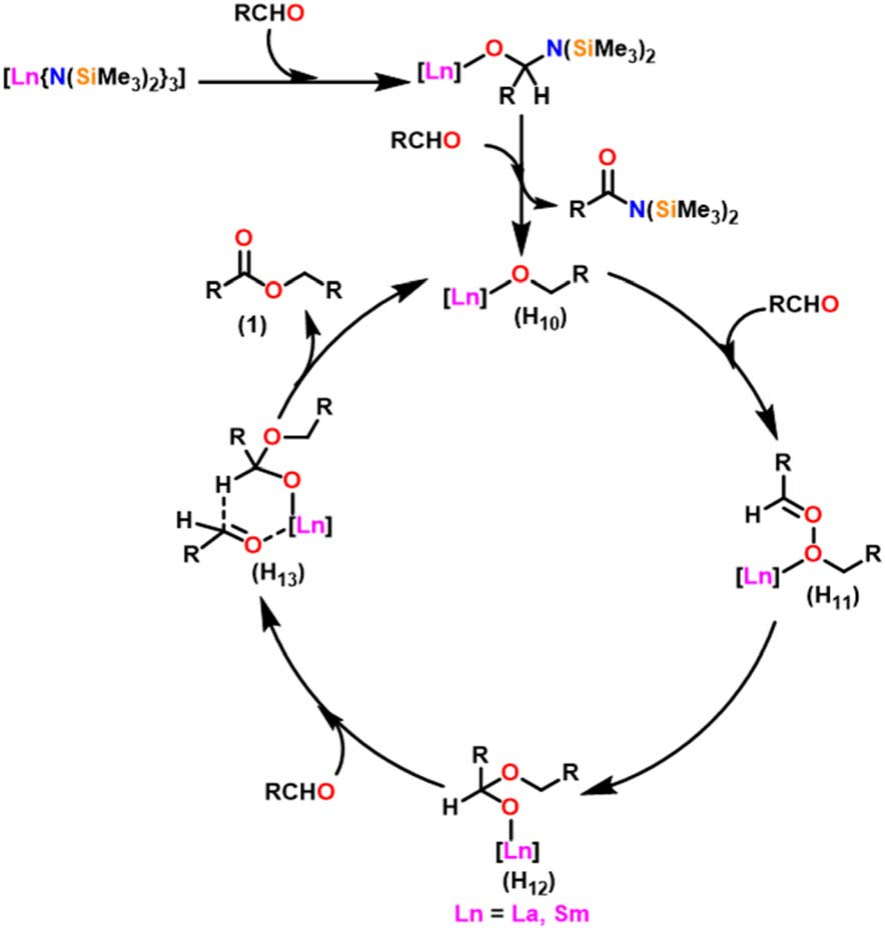
Mechanism for the lanthanide amide catalyzed Tishchenko reaction.

Berberich and co-workers reported the lanthanide-supported bis(trimethylsilyl)amides, M[N(SiMe_3_)_2_]_3_(36) [M = La (36a), Sm (36b)] as an efficient catalyst for Tishchenko reactions.^6,105^ The discovery of compound 36 has resulted in numerous advances in lanthanide chemistry. The compound 36 was recognized as a well-known starting material for lanthanide metals through the easy cleavage of the silylamide group in the past 100 years. A comparison of 36a with 36b showed that 36a displayed good catalytic activity with higher turnover frequency (TOF-87) than 36b (TOF-80). The TOF was proportional to the ionic size of the central metal atom. A comparison with other Tishchenko catalysts such as boric acid (37),^140^(35),^104^ SmI_2_(38),^42^ and [Sm{O-2,6-(^*t*^Bu)_2_-C_6_H_3_}_3_] (39)^141^ showed that complex 37 needed extreme reaction conditions whereas 35 and 38 were inert in the case of benzaldehyde and 39 displayed some catalytic activity but lower than 36a and 36b.^18^ In the Tishchenko reaction, Onozawa *et al.* described the application of the 34b complex as an active catalyst.^104^ Although the catalytic activity of 34b was lower, it competes with 36a (yield: 98% (36a), 88% (34b)). The catalyst 34b was most likely to yield the same active species as 36a, although it was much more difficult to prepare. The mechanism of the Tishchenko reaction was elucidated using ^1^H NMR spectroscopy and GC/MS studies. The lanthanide alkoxide was formed by the cleavage of N(SiMe_3_)_2_ and C_6_H_5_CON(SiMe_3_)_2_ group from the catalytically active lanthanide amide (36). The insertion of aldehyde to (H_10_) afforded the complex (H_11_) which undergoes alkoxide transfer to form (H_12_). The coordination of the second molecule of aldehyde to (H_12_) was followed by hydride migration to produce (H_13_) (Scheme 28) which further eliminated ester (1) and regenerated the (H_10_).

Catalytic and stoichiometric analyses of 36b revealed that all amide groups were eliminated without taking a significant reaction time. The ^1^H NMR spectrum revealed that the signals were associated with SmOCH_2_ groups. The catalytically active species 36a was believed to yield the same or a comparable product as 34b in a Tishchenko reaction. The following observations agree with this statement: (i) both the catalysts 36a and 34b produced similar yields, and (ii) both catalysts can interchange their original ligand shells during the process.

### 3.7. Homoleptic (3,5-di-*tert*-butyl-pyrazolate)lanthanide catalyzed Tishchenko reaction

**Scheme 29:**
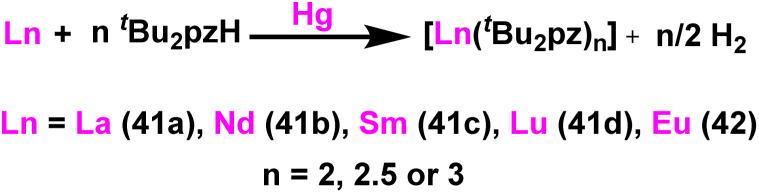
Synthesis of lanthanide pyrazolate catalysts 41–42.

Deacon *et al.* reported the highly efficient homoleptic rare-earth pyrazolate complexes such as [Sc(^*t*^Bu_2_pz)_3_] (40), [Ln_2_(^*t*^Bu_2_pz)_6_] (41) [Ln = La (41a), Nd (41b), Sm (41c), Lu (41d)], Eu_4_(^*t*^Bu_2_pz)_8_(42) and [Yb_2_(^*t*^Bu_2_pz)_5_] (43) which can acts as catalyst for the Tishchenko reaction.^18^ The complexes 41 and 42 were synthesized by the reaction of 3,5-di-*tert*-butylpyrazole (^*t*^Bu_2_pzH) with corresponding metal in the presence of mercury without any particular solvent (Scheme 29).

**Fig. 4:**
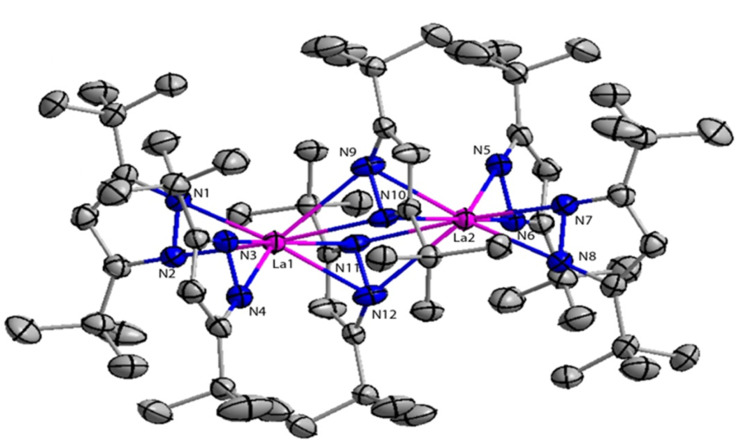
ORTEP diagram of catalyst 41a.

The structure of compound 41a is shown in Fig. 4. The angle between the La1–La2 and the bridging ligand C_3_N_2_ plane was found to be 79.2(1)°. It showed that the bridging ligand was considerably more tilted in complex 41a. The terminal La–N bond lengths for La1–N1, La1–N2, La1–N3, La1–N4 bonds were found to be 2.462(3), 2.439(3), 2.4541(3), 2.460(2) Å respectively. Terminal La–N bond lengths were slightly shorter than the bridging La1–N11 (2.593(3)) Å, La1–N12 (2.738(4)) Å bonds.

Usually, the Al(OR)_3_(44)^28,136^ were commonly used as a catalyst for the Tishchenko reaction. Instead of 44, the compound 41a can be used as a catalyst for Tishchenko reaction. The catalytic activities of compound 36a were compared to those of the highly active silylamide catalyst 41a by using a range of aliphatic aldehydes and substituted benzaldehydes. The highest production of benzyl benzoate was achieved when compound 41a was utilized while using 36a resulted in moderate yields.^105^ The product yield was higher for compound 41a than the high-speed aluminum catalysts such as [2,7-dimethyl-1,8-biphenyldioxy-bis(diisopropoxy aluminum(iii))] (44a) and [2,7-dimethyl-1,8-biphenyldioxy-bis(dibenzyloxy aluminum(iii))] (44b).^142,143^ Substrates such as aliphatic aldehydes and *o*-phthalaldehydes showed impressive quantitative yields and remarkably high TOFs when reacted with 41a, which can be compared to the results obtained with 36a. *Para*-substituted benzaldehydes were employed as substrates to determine the tolerance of 41a to functional groups. When the halogen atom was substituted to the *para* position of the benzaldehyde substrate, yields were slightly reduced. The Tishchenko reaction does not take place when the substrate contains heteroatoms such as O and S. This was because the coordination of O and S to the catalyst hinders its catalytic activity. While comparing the TOFs of benzaldehyde and substituted benzaldehydes using catalyst 41a, the latter was lower than the former. The lower reactivity of 41a than 36a was mainly due to the structural and steric factors. Complex 41a exists as a dimer in benzene-*d*_6_. After that, it comes into contact with the reaction solvent, and substrate induction, 41a dissociated into a monomer, which was a key step in the catalytic process. Although the preparation of the catalysts 41–42 was easy, these catalysts displayed less catalytic activity as compared to lanthanocene complexes.

### 3.8. Lanthanide formamidinates catalyzed Tishchenko reaction

**Scheme 30:**
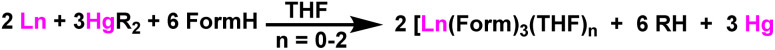
Synthesis of tris(formaminato)lanthanum(iii) complexes.

Zuyls and coworkers reported the tris(formaminato)lanthanum(iii) complexes [La(*O*-Tol-Form)_3_(THF)_2_] (45) (*O*-TolForm = *N*,*N*′-bis(*o*-tolyl)formamidinate), [La(XylForm)_3_(THF)] (46) (XylForm = *N*,*N*′-bis(2,6-dimethyl phenyl)formamidinates), and [La(EtForm)_3_] (47) (EtForm = *N*,*N*′-bis(2,6-diethylphenyl)formamidinates) as the highly efficient catalysts for Tishchenko reaction.^106^ The tris(formamidinato)lanthanide(III) complexes (45–47) were formed as a result of a reaction between lanthanide metals, *N*,*N*′-bis(aryl)formamidines (FormH), and bis(pentafluorophenyl)mercury [Hg(C_6_F_5_)_2_] in the presence of THF (Scheme 30).^144^

In previous research of the Tishchenko reaction, the rate of conversion of aldehyde to corresponding carboxylic ester was mostly dependent on the ionic radius of the lanthanide atom that was involved. The catalyst having the largest ionic radius of the central atom was said to be the most active.^105^ The catalysts 45–47, each have a distinct substituent on the aromatic ring and a unique number of THF molecules that were coordinated to the central metal atom. The catalytic activities of compounds 45 to 47 were studied using the standard reaction for the synthesis of benzyl benzoate from benzaldehyde.

^1^H NMR study indicated the formation of benzyl benzoate through an increase in the benzyl proton signal and a corresponding decrease in the aldehydic proton signal. When benzaldehyde undergoes a reaction with compound 45 in a 5 : 1 ratio, the formamidinate ligand is partially cleaved off. From the comparison of compounds 45–47, 45 exhibited a turnover of 200 h^−1^ with 99% yield within 0.5 h and possessed the highest catalytic activity. The extreme activity was a result of the smallest formamidinate ligands (which are more readily attacked by the aldehyde, hence accelerating the initial conversion), two labile THF groups, and less steric hindrance of complex 45. When compared to the other lanthanum catalysts, 40a (TOF = 87 h^−1^, yield 98%) and 34b (TOF = 1.3 h^−1^, yield 94%) displayed less activity than compounds 45–47. The catalysts (35)^104^ and (38)^42^ were commercially available catalysts for the Tishchenko reaction which showed no activity. Nevertheless, catalyst 39 (ref. 141) exhibited a certain level of activity in comparison to 35 and 38. Catalyst, [Ca{(NSiMe_3_)_2_}_2_(THF)_2_], also exhibited lower activity compared to lanthanum catalysts. Standard aluminum catalyst Al(O^i^Pr)_3_(44c)^28,136,137^ displayed a low yield (8%) of benzyl benzoate under the same conditions. Even high-speed catalysts 44a and (44b),^142,143^ or other aluminum-based catalysts^145^ for the Tishchenko reaction were not able to compete with lanthanum catalysts. The catalyst 42 failed to convert benzaldehyde to the corresponding ester. All available classical transition metal catalysts like [(C_5_H_5_)_2_ZrH_2_] (5), [H_2_Ru(PPh_3_)_2_] (6),^49^ [(C_5_H_5_)_2_HfH_2_] (48),^48^ and B(OH)_3_(49),^140^*etc.*, also failed to catalyze this reaction whereas some transition metal catalysts like amino alcohol-based iridium bifunctional catalyst^146^ gave high yields (86%) but were slower than lanthanum catalysts. Compound 45 was the best catalyst for the Tishchenko reaction rather than above mentioned catalysts. Aromatic aldehydes such as *o*-phthalaldehyde undergo intramolecular Tishchenko reaction giving high yields (quantitative) and high TOF (>1500 h^−1^). Even when utilizing 7/crown ether, the heteroaromatic aldehyde-like furfural exhibited little reactivity, and only a yield of 3.4% was achieved.^50,136,139^ In contrast, lanthanum catalysts enhanced their activity and produced much higher yields (relative to 7). Cyclic and non-cyclic aldehydes also play the role of substrates in the Tishchenko reaction. Catalysts 45–47 were slower to the Tischenko reaction of pivalaldehyde, because of the steric bulk of the starting material. And finally, it concluded that catalysts 45–47 were superior to all the catalysts discussed above. Compound 45 was able to catalyze quickly and gave high yields for aldehyde substrate with or without α-hydrogen. Butanol, when subjected to a temperature of 21 °C, generates higher coupling products such as butyl butyrate, trimeric, and tetrameric coupling products.^28,137,147,148^ These products were formed *via* tandem aldol-Tishchenko reaction, and verified by GC-MS.^24,149–155^ The extremely high activity of the catalysts 45 to 47 was attributable to the following factors: (i) Lewis acidity and (ii) ligand sphere interchangeability. These are the most active catalysts ever recorded for the Tishchenko reaction. The catalytic activity increased in the following order: 45 > 46 > 47.

Recently, Salehisaki *et al.* revealed the synthesis and applications of PhForm complexes as Tishchenko reaction catalysts.^107^ These complexes had much lower activity as compared to the known benchmark catalyst, [La(*O*-Tol-Form)_3_(THF)_2_] (45). Surprisingly, the yttrium complex from the PhForm series displayed excellent catalytic performance, contradicting previous results about lanthanide size trends. Further investigation of DMForm complexes indicated even lower activity, except [Y(DMForm)_3_(THF)] (50), which remarkably outperformed all other catalysts studied. This finding suggested that yttrium-based DMForm complexes could be appealing alternatives to the benchmark catalyst (45), particularly considering the avoidance of the carcinogenic *o*-toluidine precursor.

## 4. Anionic and cationic lanthanide complexes catalyzed Tishchenko reaction

### 4.1. Bis(amidinate) lithium lanthanide complex catalyzed Tishchenko reaction

**Scheme 31:**
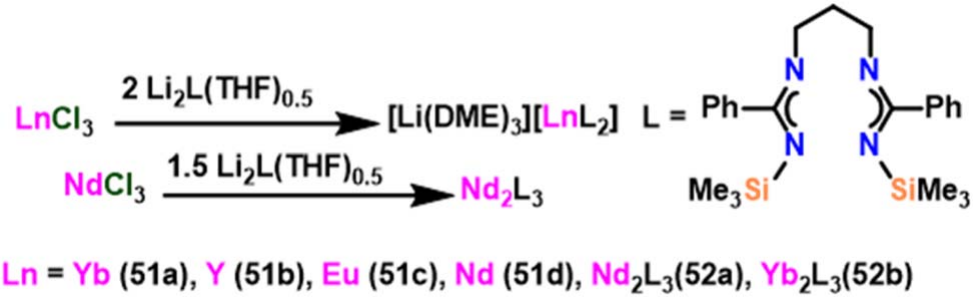
Synthesis of bis(amidinate) lithium lanthanide complexes.

Wang *et al.* introduced the ytterbium lithium bimetallic catalyst [Li(DME)_3_][LnL_2_] (51) {Ln = Yb (51a), Y (51b), Eu (51c), Nd (51d)} and [Ln_2_L_3_] (52) {Ln = Nd (52a), Yb (52b); L = [Me_3_SiNC(Ph)N(CH_2_)_3_NC(Ph)NSiMe_3_]}.^108^ The anionic complexes Eu (51c) and Nd (51d), as well as the neutral complex (52a–52b), were made by metathesis of the appropriate chloride with the lithium salt (Scheme 31).^156^ To examine the catalytic behavior of these catalysts, the Tishchenko reaction was carried out using benzaldehyde as a substrate. Unexpectedly, yields of ester were obtained after 3 hours of reaction and were found to be 37% for 51 and 25% for 52. This suggested that the catalyst used in this method was less efficient than the already reported homoleptic lanthanide amide catalyst 36.^105^ Although 51 and 52 were used as good catalysts in the amidation reaction, these catalysts failed to be efficient catalysts in the Tishchenko reaction.

### 4.2. Cationic lanthanide complex catalyzed Tishchenko reaction

**Scheme 32:**
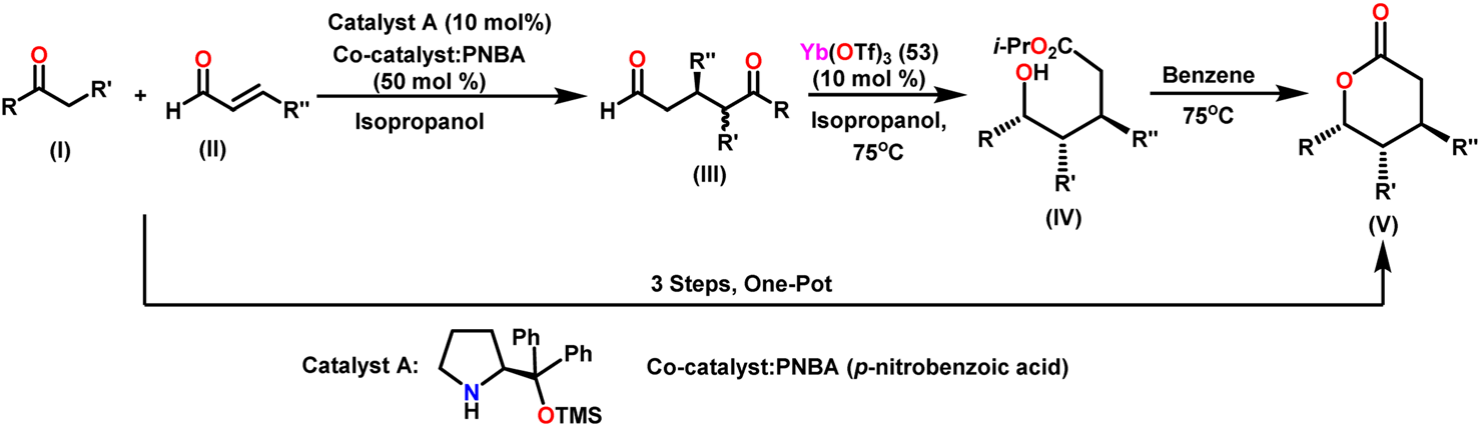
Synthesis of trisubstituted δ-lactones.

**Scheme 33:**
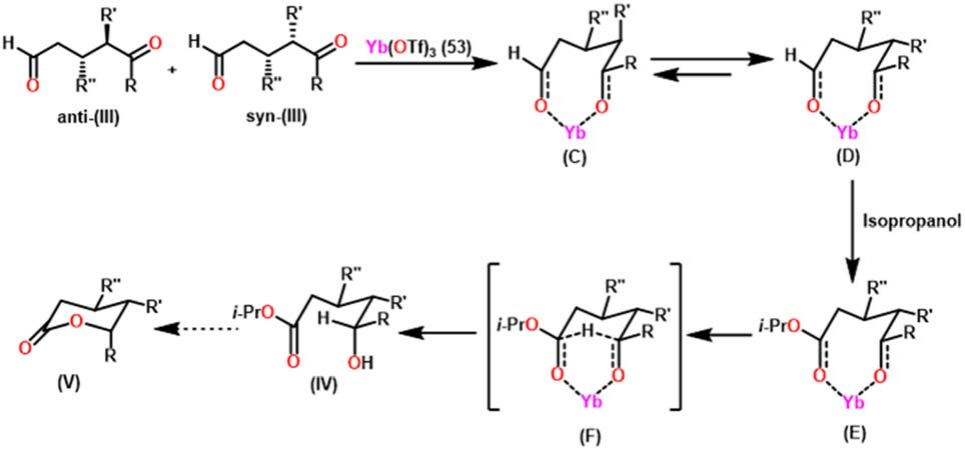
Plausible approach for transforming adducts (III) into hydroxy esters (IV).

Guevara-Pulido and colleagues gained attention for their successful one-pot synthesis of enantioenriched trisubstituted lactones, which was accomplished using a one-pot process involving sequential organocatalyzed Michael addition of ketones to enals, followed by catalytic intramolecular diastereoselective Tishchenko reaction and lactonization.^157^ Notably, the end product was a single diastereoisomer, showing that any combination of *anti* and *syn* diastereomers converted to *syn* hydroxy ester during the process. A novel approach was developed to produce trisubstituted δ-lactone from ketone and aldehyde in a single step (Scheme 32). This novel strategy decreases the requirement for purification steps, minimizes waste chemical formation, and shortens overall reaction time, increasing overall yield. In their approach, a combination of ketone (I) (1.2 equiv.), aldehyde (II) (1 equiv.), prolinol derivative A (0.1 equiv.) as a catalyst, and PNBA (0.5 equiv.) as a co-catalyst in 2-propanol was stirred at 0 °C until the reaction was complete. Following that, Yb(OTf)_3_(53) (0.1 equiv.) was added to the mixture, which was heated until (III) vanished. After extracting the 2-propanol under vacuum, the residue was dissolved in benzene and heated until (IV) was no longer detectable giving a good yield of trisubstituted δ-lactone (V). Scheme 33 describes the stereochemical outcome of the Tishchenko reaction. When a metal was coordinated to both carbonyl groups, it formed a mixture of active diastereomeric complexes (C) and (D). These complexes immediately isomerize to the most stable complex (D), as both substituents were equatorial. The addition of i-PrOH to the 5-oxopentanal-derived complex (D) took place on the exterior less hindered Re-face of the formyl group, resulting in a chelated hemiacetal complex (E). The stereochemistry at C-5 in the hydroxy ester (IV), produced as a single diastereoisomer, was determined *via* intramolecular hydride transfer across a six-membered concerted transition state (F). The final lactones (V) were produced by changing the solvent and then cyclizing (IV).

The approach performs effectively with benzyl ketones and ketosulfones as nucleophiles, and β-substituted alkyl- and aryl-enals as electrophiles. It must be emphasised that the transformation of *anti* to *syn* diastereoisomers, which began with the first Michael addition, occurs throughout the oxido-reduction step, separating lactones as the solitary diastereoisomer. This entire process is carried out in one pot, resulting in less waste, time, and no need for purification procedures.

## 5. Grafted lanthanide complexes catalyzed Tishchenko reaction

### 5.1. Grafted lanthanide amides complexes catalyzed Tishchenko reaction

**Scheme 34:**
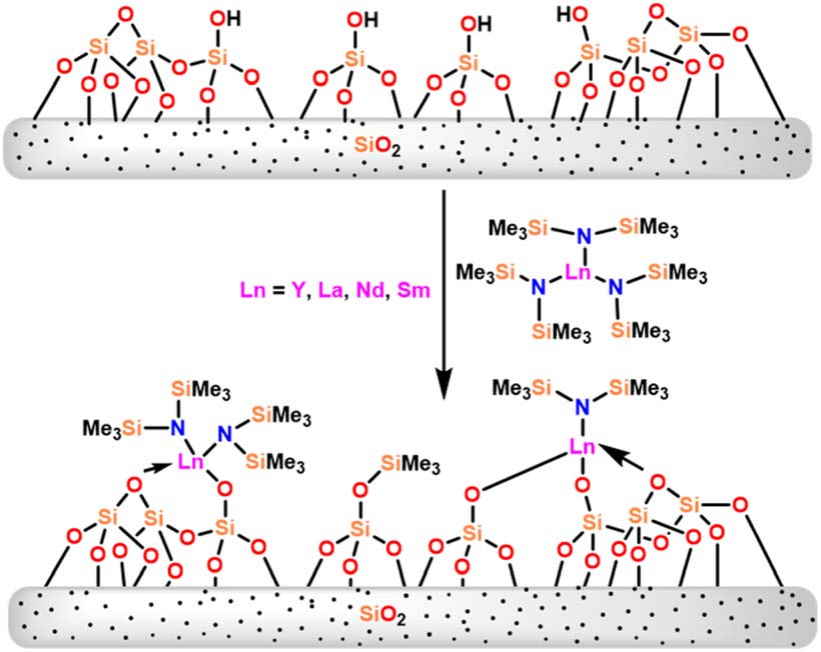
Reaction of lanthanide amide 36 with silica surface.

Catalysts 36a and 36b were found to be superior to the metallocene [(C_5_Me_5_)_2_YCH(SiMe_3_)_2_] (34c) in terms of quantitative yields for the hydroamination/cyclization of amino alkynes. The comparison of the catalytic activity of 36 with grafted amides (silica-supported yttrium Y__700_ (54a) and samarium Sm__700_ (54b)) was carried out by Gauvin and the group.^109^ The grafted lanthanide catalysts (54a–54b) were prepared by reaction of 36 with the silica surface (Scheme 34).

In order to compare the catalytic activity, the benzaldehyde–benzyl benzoate reaction was used as a model reaction. For catalysts 54a and 54b, a conversion of benzaldehyde was 31 and 57 equivalents respectively. After the separation of the supernatant, no activity was seen after 15 h of benzaldehyde addition. It indicated that the active species exists on the surface of silica. For trisamido catalysts, 36b and 36c the conversion of aldehydes was higher 89% and 67% respectively. It showed that supported catalysts were found to be less efficient than unsupported ones (36b and 36c) under the same reaction conditions. The yield reached a plateau after 6 h with grafted catalysts 54a and 54b. This was due to the inhibition process of the product formed by the reaction. This behavior of the catalysts was attributed to the distinct natures of the active sites. In homogeneous lanthanide clusters, benzylato ligands were small to form monomeric complexes,^158^ whereas surface lanthanide alkoxides were mononuclear in grafted catalysts. After 3 hours of reaction time, the supernatant was removed from the solid catalyst. The reaction-modified silica was rinsed with toluene, and then a fresh substrate was added to the solution. Conversion by the first run was 38% whereas the second and third run was 16% and 15% respectively. The catalytic activity of 54 was low compared to 36.

### 5.2. Hybrid material [SBA-15]Sm[N(SiMe_3_)_2_]_*x*_ catalyzed Tishchenko reaction

**Scheme 35:**
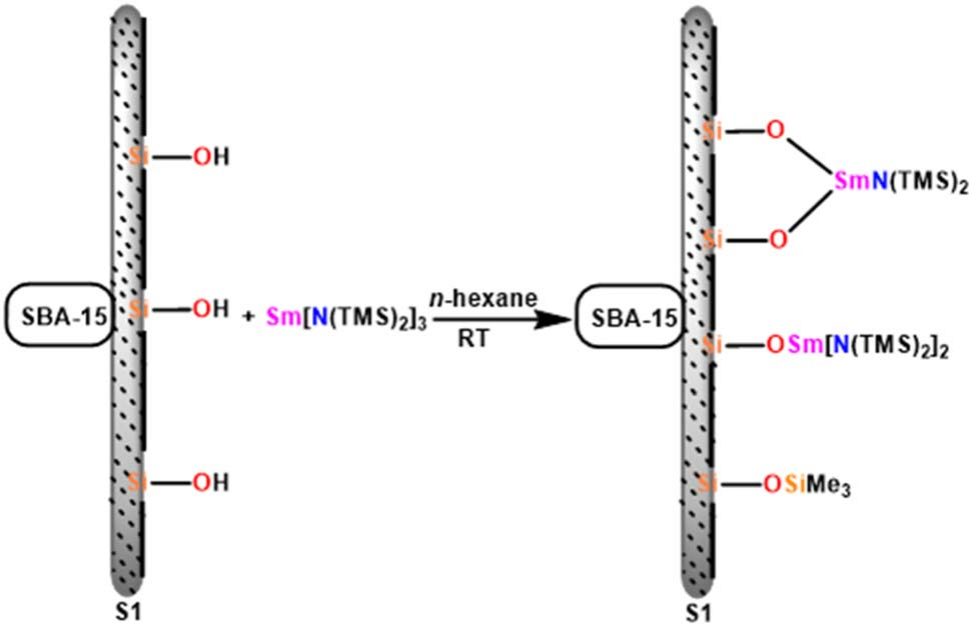
Immobilization of Sm[N(SiMe_3_)_2_]_3_ onto mesoporous SBA-15.

Chen and team delved into the catalytic properties of a novel hybrid material [SBA-15]Sm[N(SiMe_3_)_2_]_*x*_, which was synthesized by incorporating the active Sm[N(SiMe_3_)_2_]_3_(36b) into a novel mesoporous material SBA-15, known for its two-dimensional hexagonal pore structure. [SBA-15]Sm[N(SiMe_3_)_2_]x (55) is an efficient and mild catalyst for the Tishchenko reaction that converts benzaldehyde to benzyl benzoate.^110^ Compound 55 was prepared using the immobilization method. It was easily carried out by mixing mesoporous SBA-15 with 36b in hexane for a few days at room temperature (Scheme 35).^159–162^

**Scheme 36:**
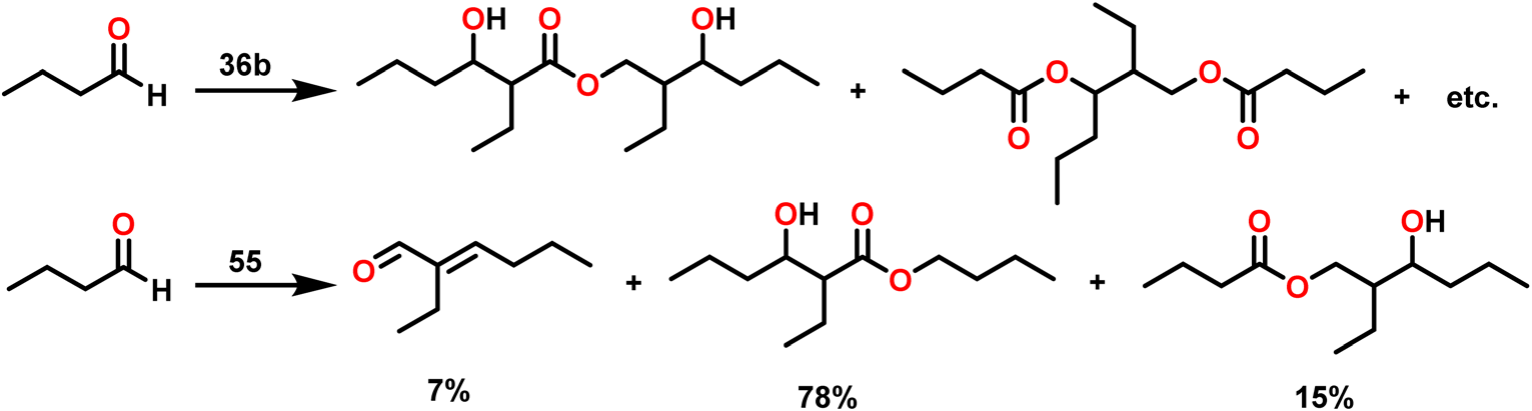
The reaction of butyl aldehyde catalyzed by 36b and 55.

**Scheme 37:**
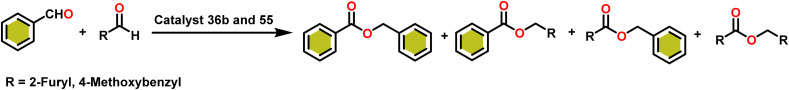
Mixed Tishchenko reaction catalyzed by 36b and 55.

By comparing the catalysts 36b and 55, the reaction rate of heterogeneous catalyst 55 was somewhat slower than homogeneous catalyst 36b, because of the restricted diffusion. Under the same experimental conditions yields were also lower than the corresponding homogeneous catalyst 36b. However, the overall yield of 55 was comparable with the homogeneous catalyst with prolonged reaction time. In the case of substituted aldehydes, low yields were obtained as a result of the steric hindrance and the +M effect of the substituent on the phenyl ring. If butyl aldehyde was used as a substrate with 55 as a catalyst, different product selectivity could be achieved rather than a homogeneous 36b catalyst. Trimers and oligomers were obtained while using 55 and 36b respectively (Scheme 36). The difference between the above was the pore confinement of hybrid material, which did not allow the trimeric substance to undergo further aggregation with other aldehydes. Catalyst 55 showed higher stability towards oxygen than 36b. This implied that by utilizing 55 as a catalyst, the reaction can be carried out without the need for N_2_ protection, which was not achievable with 36b. The catalyst 55 can be removed easily from the reaction mixture and reused. In many cases recovered material showed inherent activity while using subsequent runs. However, X-ray diffraction and N_2_ sorption examination demonstrated that the recovered material possesses the same pore size and well-ordered microstructure as 55. These findings indicated that there was no obstruction of pores in 55 during the whole catalytic process, perhaps because of the significant pore width and volume of SBA-15. The elemental analyses and IR studies suggested that silylamide ligands were replaced by formed alkoxide ligands.^104^ To investigate the catalytic performance of hybrid catalyst 55, the mixed Tishchenko reaction was conducted (Scheme 37). When various types of aldehydes were combined in equal proportions (1 : 1) with hybrid catalyst 55, the formation of the resulting cross-products was greater compared to the homogeneous catalysts. This selectivity resulted from the spatial restriction and diffusion control of the surface confinement of the hybrid solid material.

## 6. Lanthanide heterometal clusters catalyzed Tishchenko reaction

### 6.1. Lanthanide homogeneous heterometal clusters catalyzed Tishchenko reaction

Li *et al.* reported that a highly effective catalyst for the Tishchenko reaction can be synthesized using heterometal alkoxide clusters of group 3 metals and lanthanides with Ln_2_Na_8_(OCH_2_CH_2_NMe_2_)_12_(OH)_2_(56) [Ln = Yb (56a), Pr (56b), Nd (56c), Sm (56d), and Y (56e)].^111,163^ The reaction of benzaldehyde with 1 mol% of 56b at room temperature for approximately 2 hours afforded 78% yield of the corresponding ester. The catalytic activities of all the lanthanide heterometal clusters 56a–56e were tested and all gave good yields. Compound 56a exhibited superior activity with a 93% isolated yield, while 56d displayed the lowest yield and lower activity. In the cases of NaOCH_2_CH_2_NMe_2_(57), there was no reaction, which means that benzaldehyde did not undergo a Tishchenko reaction without the lanthanide complex. Despite being a lanthanide complex, Nd(OCH_2_CH_2_NMe_2_) (58) was inactive for the Tishchenko reaction. 56b has a catalytic activity that rivals other high-speed catalysts, such as 44a and 44b^142^ in the Tishchenko reaction. The activity of 56b exceeded that of 35,^6,104^38,^6,105^ and 39 (ref. 6) but was lower than 36. The only benefit of 56b was its reduced susceptibility to air and moisture. Several kinds of aromatic, heteroaromatic, and aliphatic aldehydes were examined with 56b. All reactions were smoothly taking place with 1 mol% of 56b except for 2-furaldehyde (39% yield) afforded moderate to higher yields. The lower yield of 2-furaldehyde resulted from the coordination of each oxygen in the ether to the lanthanide metal, which resulted in the formation of a stable five-membered metallocycle that partially inhibits the active species. This was the same situation as with lanthanide silylamide complexes.^6,105^

Tolerance of 56b was tested with different types of *para*-substituted benzaldehyde substrates. According to the results, cluster 56b was very tolerant to functional groups, and a higher yield was produced when the aromatic ring contained an electron-donating substituent. This was due to the coordination of metal with the carbonyl group. Ultimately, it was discovered that heterometal alkoxide clusters 56a–56e outperformed previously documented lanthanide silylamide catalysts in the Tishchenko reaction. This was attributed to their enhanced ability to withstand a wide range of functional groups. This outcome could be attributed to the cooperation between the lanthanide and sodium centers. Various types of lanthanide catalysts and their catalytic activities, yield, and TOFs are described in Table 8.

**Table 8:** List of lanthanide catalysts used for Tishchenko reaction of benzaldehyde to benzyl benzoate

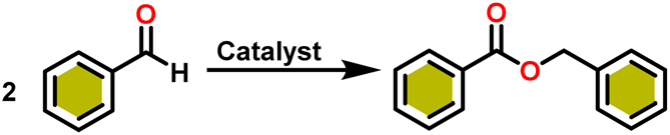							
S. no.	Catalyst	TOF [h^−1^]	Yield [%]	S. no.	Catalyst	TOF [h^−1^]	Yield [%]
1	EtPrI (33a)	—	32	17	[La(XylForm)_3_(thf)] (46)	133	93
2	EtNdI (33b)	—	40	18	[La(EtForm)_3_] (47)	100	99^a^
3	EtSmI (33c)	—	30	19	[Y(DMForm)_3_(THF)] (50)	—	98
4	Cp*_2_NdCH(SiMe_3_)_2_(34a)	—	88	20	[Li(DME)_3_][YbL_2_] (51a)	—	37
5	Cp*_2_LaCH(SiMe_3_)_2_(34b)	1.3	94		{L = [Me_3_SiNC(Ph)N(CH_2_)_3_NC(Ph)NSiMe_3_]}		
6	[La(O^*i*^Pr)_3_] (35)	—	—	21	[Yb_2_L_3_] (52b)	—	25
7	La[N(SiMe_3_)_2_]_3_(36a)	87	98		{L = [Me_3_SiNC(Ph)N(CH_2_)_3_NC(Ph)NSiMe_3_]}		
8	Sm[N(SiMe_3_)_2_]_3_(36b)	80	98	22	Sm__700_(54b)	—	—
9	[Sm{O-2,6-(^*t*^Bu)_2_-C_6_H_3_}_3_] (49)	1.9	70	23	Yb_2_Na_8_(OCH_2_CH_2_NMe_2_)_12_(OH)_2_(56a)	—	93
10	[La_2_(^*t*^Bu_2_pz)_6_] (41a)	4.2	Quant.	24	Pr_2_Na_8_(OCH_2_CH_2_NMe_2_)_12_(OH)_2_(56b)	—	75
11	[Nd_2_(^*t*^Bu_2_pz)_6_] (41b)	—	—	25	Nd_2_Na_8_(OCH_2_CH_2_NMe_2_)_12_(OH)_2_(56c)	—	83
12	[Sm_2_(^*t*^Bu_2_pz)_6_] (41c)	—	—	26	Sm_2_Na_8_(OCH_2_CH_2_NMe_2_)_12_(OH)_2_(56d)	—	69
13	[Lu_2_(^*t*^Bu_2_pz)_6_] (41d)	—	—	27	[SBA-15]Sm[N(SiMe_3_)_2_]_*x*_(55)	—	80 (1st run)
14	Eu_4_(^*t*^Bu_2_pz)_8_(42)	—	—				70 (2nd run)
15	Yb_2_(^*t*^Bu_2_pz)_5_(43)	—	—				50 (3rd run)
16	[La(*o*-Tol-Form)_3_(thf)_2_] (45)	200	99^a^				

a Determined by ^1^H NMR spectroscopy (TMS as Internal standard).

The Tishchenko reaction involves the conversion of different types of aldehyde substrates, including aromatic, heteroaromatic, and aliphatic aldehydes, into corresponding esters. This transformation is facilitated by the use of various lanthanide complexes (33–56) as catalysts, as shown in Table 9.

**Table 9:** Results for the dimerization of various aldehydes using different lanthanide catalysts

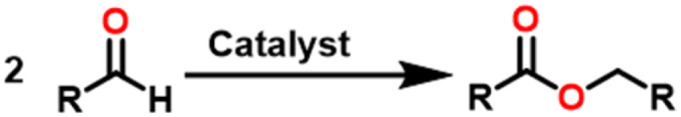							
S. no.	Catalyst	RCHO	Yield (%)	S. no.	Catalyst	RCHO	Yield (%)
1	33a	Benzaldehyde	32	37	34b	4-Chlorobenzaldehyde	89
2	33b	Benzaldehyde	40	38	36a	4-Chlorobenzaldehyde	47
3	33c	Benzaldehyde	30	39	36b	4-Chlorobenzaldehyde	85
4	34a	Benzaldehyde	88	40	41a	4-Chlorobenzaldehyde	22^a^
5	34b	Benzaldehyde	94	41	55	4-Chlorobenzaldehyde	82, 80 and 81 (1^st^, 2^nd^ and 3^rd^ runs respectively)
6	36a	Benzaldehyde	98	42	56a	4-Chlorobenzaldehyde	74
7	36b	Benzaldehyde	85	43	34b	4-Cyanobenzaldehyde	96
8	41a	Benzaldehyde	Quant.	44	35	4-Cyanobenzaldehyde	70
9	50	Benzaldehyde	98	45	36a	4-Cyanobenzaldehyde	80
10	51a	Benzaldehyde	37	46	56a	4-Cyanobenzaldehyde	76
11	52b	Benzaldehyde	25	47	33b	4-Methylbenzaldehyde	40
12	56a	Benzaldehyde	93	48	34b	4-Methylbenzaldehyde	88
13	56b	Benzaldehyde	75	49	36a	4-Methylbenzaldehyde	78
14	56c	Benzaldehyde	83	50	36b	4-Methylbenzaldehyde	82
15	56d	Benzaldehyde	69	51	55	4-Methylbenzaldehyde	78, 75 and 54 (1^st^, 2^nd^ and 3^rd^ runs respectively)
16	57	Benzaldehyde	No reaction	52	33b	4-Methoxybenzaldehyde	11
17	58	Benzaldehyde	No reaction	53	34b	4-Methoxybenzaldehyde	95
18	55	Benzaldehyde	80, 70 and 50 (1^st^, 2^nd^ and 3^rd^ runs respectively)	54	36a	4-Methoxybenzaldehyde	86
19	34b	Cyclohexanecarbaldehyde	95	55	56a	4-Methoxybenzaldehyde	90
20	35	Cyclohexanecarbaldehyde	84	56	34a	Thiophene-2-carbaldehyde	31
21	36a	Cyclohexanecarbaldehyde	80	57	34b	Thiophene-2-carbaldehyde	60
22	41a	Cyclohexanecarbaldehyde	Quant.^a^	58	45	Thiophene-2-carbaldehyde	88
23	45	Cyclohexanecarbaldehyde	Quant.	59	47	Thiophene-2-carbaldehyde	88
24	46	Cyclohexanecarbaldehyde	Quant.	60	33b	Pivalaldehyde	37
25	47	Cyclohexanecarbaldehyde	Quant.	61	34a	Pivalaldehyde	46
26	56a	Cyclohexanecarbaldehyde	81	62	34b	Pivalaldehyde	Quant.
27	34b	2-Furaldehyde	77	63	36a	Pivalaldehyde	80
28	36a	2-Furaldehyde	40	64	41a	Pivalaldehyde	96
29	36b	2-Furaldehyde	45	65	45	Pivalaldehyde	91
30	45	2-Furaldehyde	79	66	46	Pivalaldehyde	90
31	56a	2-Furaldehyde	39	67	47	Pivalaldehyde	86
32	55	2-Furaldehyde	30, 30 and 16 (1^st^, 2^nd^ and 3^rd^ runs respectively)	68	36a	Butyraldehyde	45
33	34b	4-Fluorobenzaldehyde	95	69	45	Butyraldehyde	16^b^
34	36a	4-Fluorobenzaldehyde	66	70	36b	2,3-Dimethoxybenzaldehyde	10
35	41a	4-Fluorobenzaldehyde	87^a^	71	55	2,3-Dimethoxybenzaldehyde	5
36	33b	4-Chlorobenzaldehyde	40				

a Conversion in NMR scale.

b Trimers and tetramers were produced as a by-product determined by GC-MS.

## 7. Conclusion

This review meticulously examines actinide and lanthanide complexes as potential Tishchenko reaction catalysts. Their advantages, including high yields, broad substrate scope, and tolerance to functional groups, identify them as strong contenders for future dominance in Tishchenko applications. Employing specifically designed ligands and optimized reaction conditions can circumvent challenges encountered during the Tishchenko reaction, enabling organoactinide and organolanthanide complexes to excel as powerful catalysts for the esterification of aldehydes. Ligand design, specifically tuning their steric and electronic features, is a key strategy for optimizing the catalytic activity and selectivity of organoactinide and organolanthanide complexes. The ligands imidazolin-2-imine, benzimidazolin-2-imine, and perimidin-2-imine have shown remarkable activity and selectivity in the Tishchenko reaction, and have emerged as extremely effective scaffolds for organoactinide catalysts. The Tishchenko reaction mechanism with organoactinide catalysts involves many phases, including aldehyde insertion into the metal–carbon bond, rate-determining hydride transfer, and ester elimination. Lanthanide catalysts also perform a similar Tishchenko reaction, involving aldehyde insertion, hydride migration, and alkoxide transfer.

In this review, we explored the different types of lanthanide complexes, including ethyl lanthanoid iodides, lanthanocene complexes, lanthanide amides, homoleptic (3,5-di-*tert*-butyl-pyrazolate) lanthanides, lanthanide formamidinates, and bis(amidinate) lithium lanthanides. Ease of separation and reusability make grafted lanthanide catalysts such as silica-supported Y__700_ and Sm__700_ promising lanthanide catalysts for practical applications.

The mechanism of the Tishchenko reaction with actinide and lanthanide catalysts is still not fully understood. Further research is required to determine the precise functions of the actinide and lanthanide metals, as well as the ligands, in the catalytic cycle. Understanding the mechanism allows for more efficient catalyst design. The strategic application of organoactinide and organolanthanide complexes offers novel opportunities for the utilization of actinides and lanthanides in organic synthesis. The use of specifically designed ligand designs and reaction conditions can enable the synthesis of more active organoactinide and organolanthanide catalysts. The Tishchenko reaction is still being researched, and new actinide and lanthanide-based catalysts are being synthesized all the time. It would be fascinating to examine how the catalysts detailed here compare to the most recent breakthroughs in the field.

## Conflicts of interest

There are no conflicts to declare.

## Supplementary Material
